# Identification of key metabolic changes during liver fibrosis progression in rats using a urine and serum metabolomics approach

**DOI:** 10.1038/s41598-017-11759-z

**Published:** 2017-09-12

**Authors:** Hong Chang, Hong-yu Meng, Shu-min Liu, Yu Wang, Xiao-xu Yang, Fang Lu, Hong-yu Wang

**Affiliations:** 1 Chinese Medicine Toxicological Laboratory, Heilongjiang University of Chinese Medicine, Harbin, P.R. China; 2 School of Pharmacy, Baotou Medical College, Inner Mongolia, Baotou, P.R. China; 3 Drug Safety Evaluation Center, Heilongjiang University of Chinese Medicine, Harbin, P.R. China

## Abstract

Reversibility of hepatic fibrosis is an intrinsic response to chronic injury, and with on-going damage, fibrosis can progress to its end-stage consequence, cirrhosis. Non-invasive and reliable biomarkers for early detection of liver fibrosis are needed. Based on the CCl_4_-induced liver fibrosis rat model, urinary and serum metabolic profiling performed by LC-QTOF-MS associated with histological progression were utilized to identify liver fibrosis-specific potential biomarkers for early prediction and to reveal significant fibrotic pathways and their dynamic changes in different stages of liver fibrosis. Finally, nine differential metabolites in urine and ten in serum were selected and identified involving the most relevant metabolic pathways. Perturbations of tryptophan, valine, leucine, isoleucine, and citrate (TCA) cycle metabolites, along with sphingolipid and glycerophospholipid metabolites, occurred from the onset of liver fibrosis. Furthermore, dysregulation of valine and bile acid biosynthesis metabolites occurred in the intermediate and advanced stages. More importantly, among these metabolites, urinary kynurenic acid, 5-hydroxyindoleacetyl glycine, 4-(2-amino-3-hydroxyphenyl)-2,4-dioxobutanoic acid and serum sphinganine, sphingomyelin,﻿ L-leucine, L-tryptophan, and LysoPC(17:0) changed at all time points and may serve as potential early biomarkers for the diagnosis of hepatic fibrosis and as therapeutic targets. Overall, this work evaluates the potential of these metabolites for the early detection of liver fibrosis.

## Introduction

Hepatic fibrosis results from a dynamic process regarded as the result of extracellular matrix (ECM) accumulation following liver injury^1^. With protracted liver damage, fibrosis may progress towards its irreversible end-stage consequence, cirrhosis, the leading cause of liver-disease-related morbidity and mortality worldwide^2, 3^. Nonetheless, due to the liver’s capacity to regenerate, fibrosis can be reversed^1^. Therefore, early detection and regression of hepatic fibrosis are urgently needed.

Currently, evaluation of hepatic fibrosis is primarily based on a liver biopsy (LB), which is considered the golden standard for diagnosis. However, the invasive nature of this procedure make it risky and prone to considerable sampling error and assessment variability^4^. Accordingly, progress in validating new region-specific and accurate non-invasive biomarkers is urgently needed to accurately assess and diagnose the early stages of hepatic fibrosis.

Metabolomics is the latest systems biology technique for identification of global metabolic profile changes of endogenous substances in biological systems and may be used to characterize the different physiological and pathological states of organisms under external physical, chemical and environmental stimuli^5, 6^. Recently, metabolomics has been extensively used in the diagnosis and monitoring of disease progression, providing crucial insights into disease pathogenesis^7^. The main methodologies used for metabonomics analysis are mass spectrometry (MS) and nuclear magnetic resonance spectroscopy^8^. In MS-based metabolomics, ultra-performance liquid chromatography–quadrupole time-of-flight high-definition mass spectrometry (UPLC-QTOF/HDMS), is regarded as one of the best analytical techniques due to its high analytic speed and sensitivity and high resolution of chromatographic peaks for complex biological samples^9^. Investigators currently employ this technique to investigate diagnostic and potential disease-related biomarkers^10, 11^. At present, a number of metabolomic studies have reported metabolite changes in a variety of liver disease including hepatocellular carcinoma, non-alcoholic fatty liver disease (NAFLD), and alcoholic hepatitis (AH)^12–16^. Metabolomics has also been applied to develop diagnostic or prognostic biomarkers for liver fibrosis. For example, Huang, H *et al*. identified a set of biomarkers (lysophosphatidylcholines *et al*.) to discriminate early liver inflammation and fibrosis stages in patients with chronic hepatitis B^4^. Tokushige, K *et al*. found some biomarkers suggesting that disturbance of hormone metabolism was associated with the progression of fibrosis in NAFLD^14^.

Animal models of human diseases play important roles in the study of pathogenic processes due to easy management and obviation of clinical sampling and financial or ethical problems^17^. The hepatic fibrosis model induced by carbon tetrachloride (CCl_4_) in rats is well-characterized^18^. Repeated injections of CCl_4_ cause liver damage, inflammation and fibrosis in rats that are clinically similar to the human pathology of liver fibrosis, as assessed by serum analysis and histological staining^19^. To data, this model has been used for the elucidation of the mechanism of underlying the pathogenesis of liver fibrosis and diagnostic biomarker discovery^20–22^. Nevertheless, these studies have limited value in identifying changes in biomarkers of hepatic fibrosis and for monitoring the entire development and disease progression process. Additionally, few of studies have investigated the combination of urine and serum metabolomics profiling, which can provide more comprehensive insights into the pathogenesis of liver fibrosis.

In the present study using the CCl_4_-induced liver fibrosis rat model, ultra-performance liquid chromatography quadrupole time of flight mass spectrometry (UPLC-QTOF-MS)-based metabolomics was performed to develop a serum and urinary metabolite profile, to identify key metabolic changes during liver fibrosis progression, and to clarify fibrosis progression and provide diagnostic information for the early detection of hepatic fibrosis.

## Results

### Histopathological observations

**Figure 1:**
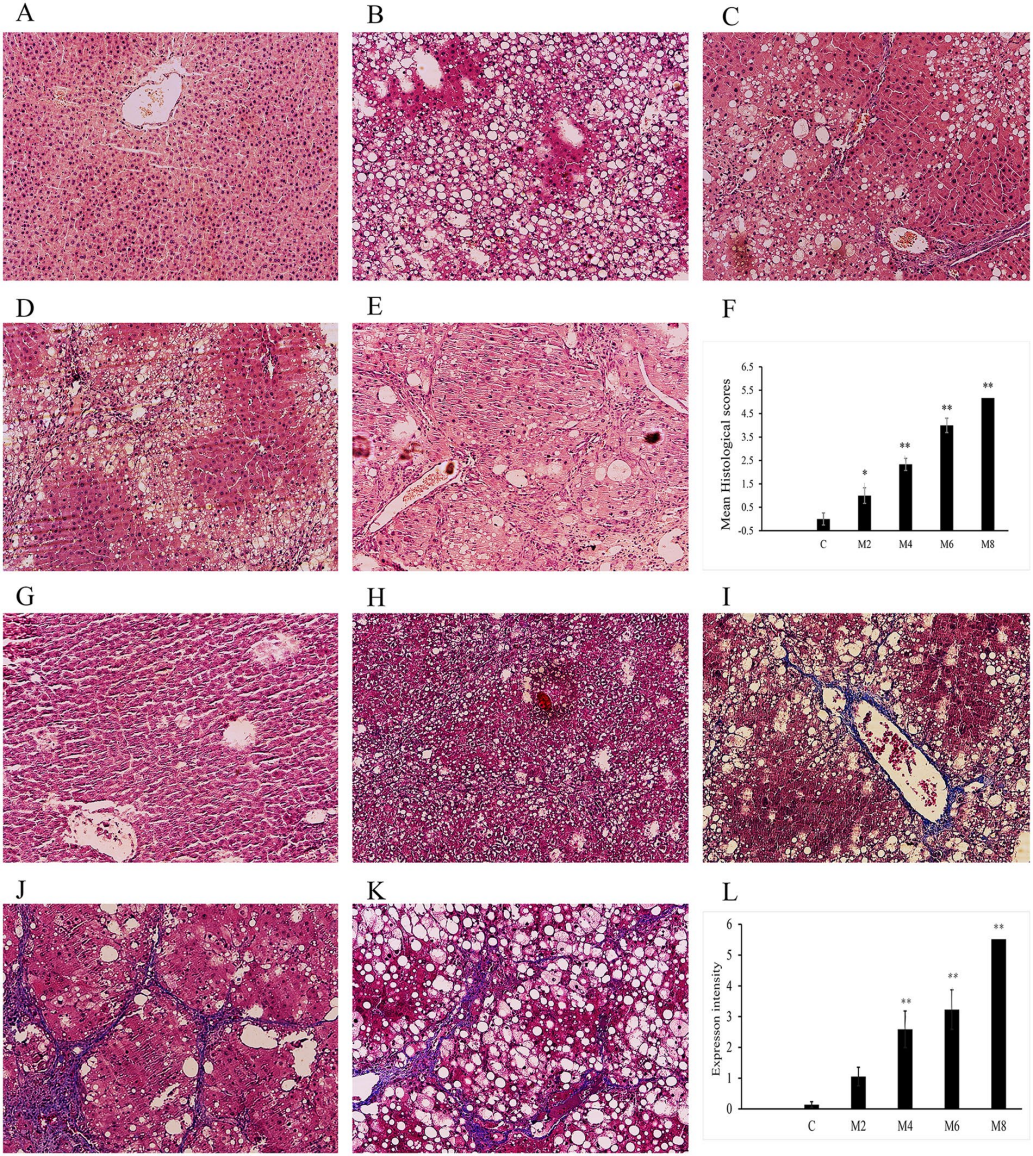
Representative photomicrographs of the H&E and Masson’s trichrome stainings (100-fold). (**A–E**) Liver tissues were stained using H&E in the control and model groups at weeks 2, 4, 6 and 8. (**G–K**) Liver tissues were stained using Masson staining in the control and model groups at weeks 2, 4, 6 and 8. All values are presented as means ± SD. Statistical significance was calculated using the two-tailed Student’s t-test with a 95% CI. *P < 0.05, **P < 0.01 compared to the control group.

Figure 1 shows representative photomicrographs of the haematoxylin & eosin (H&E) and Masson’s trichrome-stained liver tissues from the control and model groups at weeks 2, 4, 6 and 8. H&E staining (Fig. 1A–F) indicated the control group showed a normal lobular architecture with central veins and radiating hepatic cords. For the model group, there was a series of severe changes in liver morphology, including progressive degrees of fatty metamorphosis, inflammation and necrosis. By week 2, no significant abnormalities, but some fatty metamorphoses, were found in the livers of the model rats. In contrast, significant pathological injuries occurred, including slight granular degeneration, inflammatory cell infiltration and an increase in fibroblasts between the portal area and the interlobular area from 4 to 6 weeks. At week 8, the rats showed further accumulated dead or ballooned hepatocytes, an increased number of fibroblasts and hepatic lobe reconstruction. Additionally, Masson’s trichrome staining (Fig. 1G–L) indicated collagen deposition and fibrosis accumulation in the CCl_4_-injected rats at weeks 4, 6 and 8; whereas no morphological changes were observed in the control or model group rats at week 2.

### Trajectory and metabolic profiling analysish

**Figure 2:**
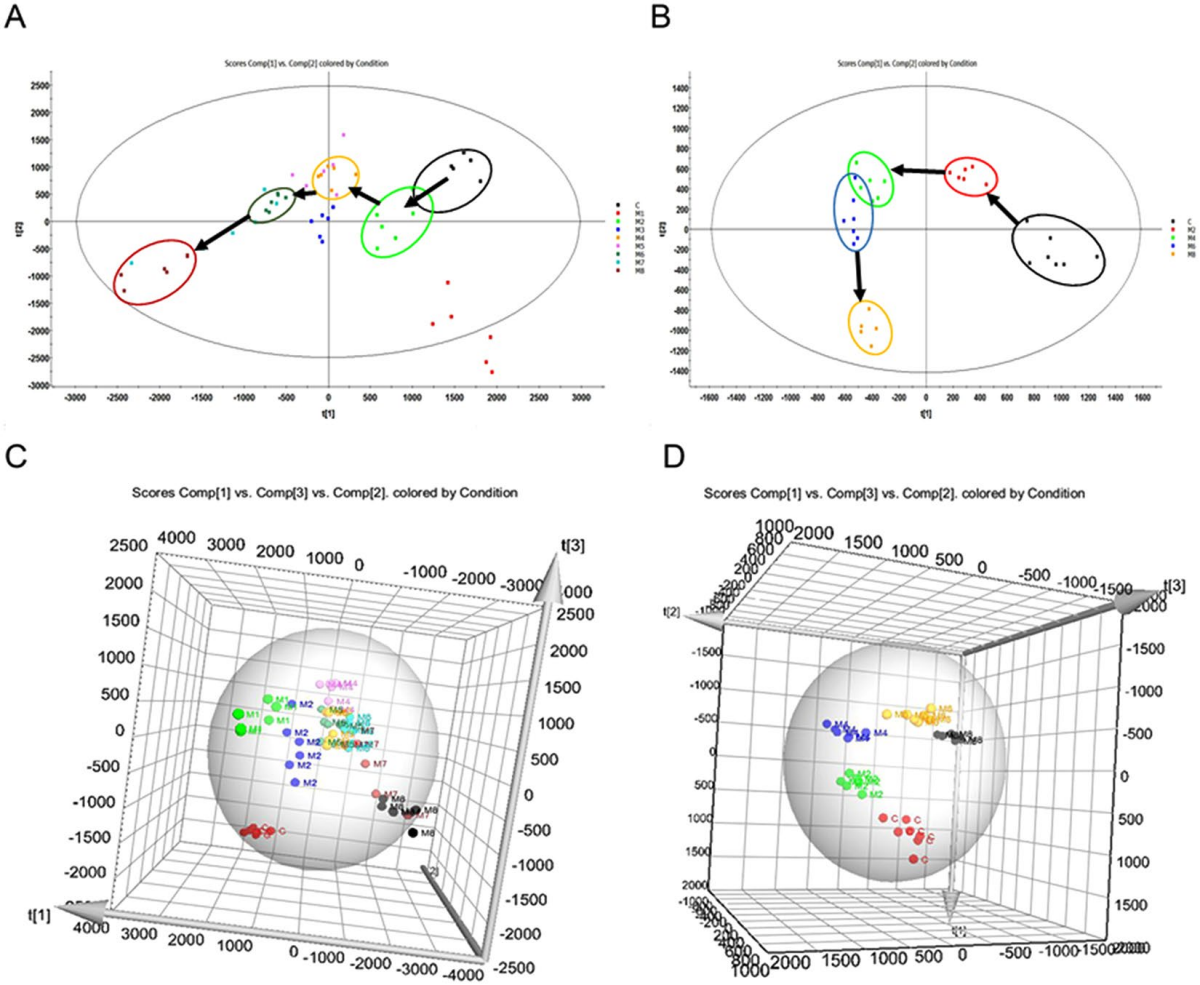
Trajectory analysis of PLS-DA and 3D PLS-DA score plots for liver fibrosis. (**A,C**) Urine samples in positive mode (R^2^Y = 86%, Q^2^ = 62%). (**B,D**) Serum samples in positive mode (R^2^Y = 98%, Q^2^ = 93%).

Metabolic profiles of urine and serum samples were acquired using UPLC-QTOF/HDMS in the positive and negative ion modes. Typically, the trajectory analysis of the principal components analysis (PCA) score plots for the liver fibrosis at different time points showed clear separations in both positive (Fig. 2) and negative ion modes (Supplementary Fig. S1). The serum and urine parameters (R^2^Y, Q^2^) used for the classification from the Waters EZinto 3.0 software exhibited good fitness and prediction, respectively. The tracks of the urine and serum metabolic profiles clearly demonstrated time-dependent changes and reflected changes at different evolutionary stages of liver fibrosis (arrows indicate the variable trends in the metabolic patterns). Furthermore, the urinary metabolic profiles between the control and model groups at week 8 clearly showed that maximum separation caused significant metabolic disturbances and pathobiological changes as a result of liver fibrosis, especially in the positive mode. To further validate the separation of the metabolic profiles at different evolutionary stages of liver fibrosis, an orthogonal partial least squares discriminant analysis (OPLS-DA) was established. The control group and liver fibrosis model group could be separated completely at all time points (weeks 2, 4, 6, and 8) in the OPLS-DA score plots (Supplementary Fig. S2A–D). The parameters (R^2^X, R^2^Y and Q^2^) for each model are shown in Supplementary Table S1, which indicates that these models have satisfactory goodness-of-fit and goodness-of-prediction.

### Selection and identification of important differential metabolites

**Figure 3:**
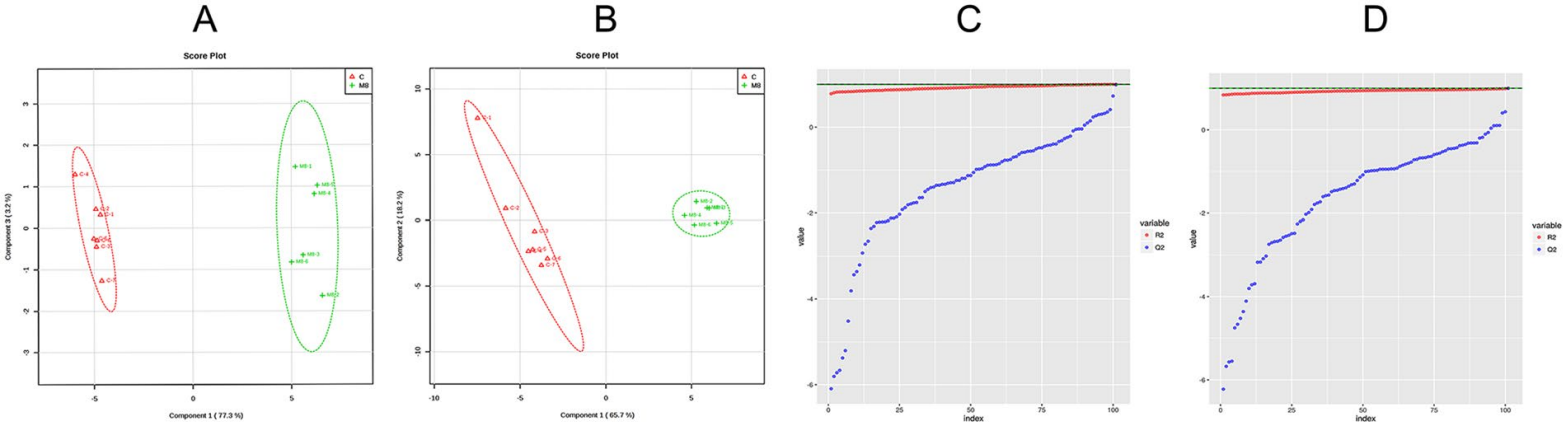
PLS-DA score plots of urine (**A**) and serum (**B**) identified differential metabolits in the control and model groups at week 8. Validation of their urine (**C**) and serum (**D**) PLS-DA models by class permutation tests at week 8. All these models were analysed by OmicsBean (http://www.omicsbean.cn/).

**Figure 4:**
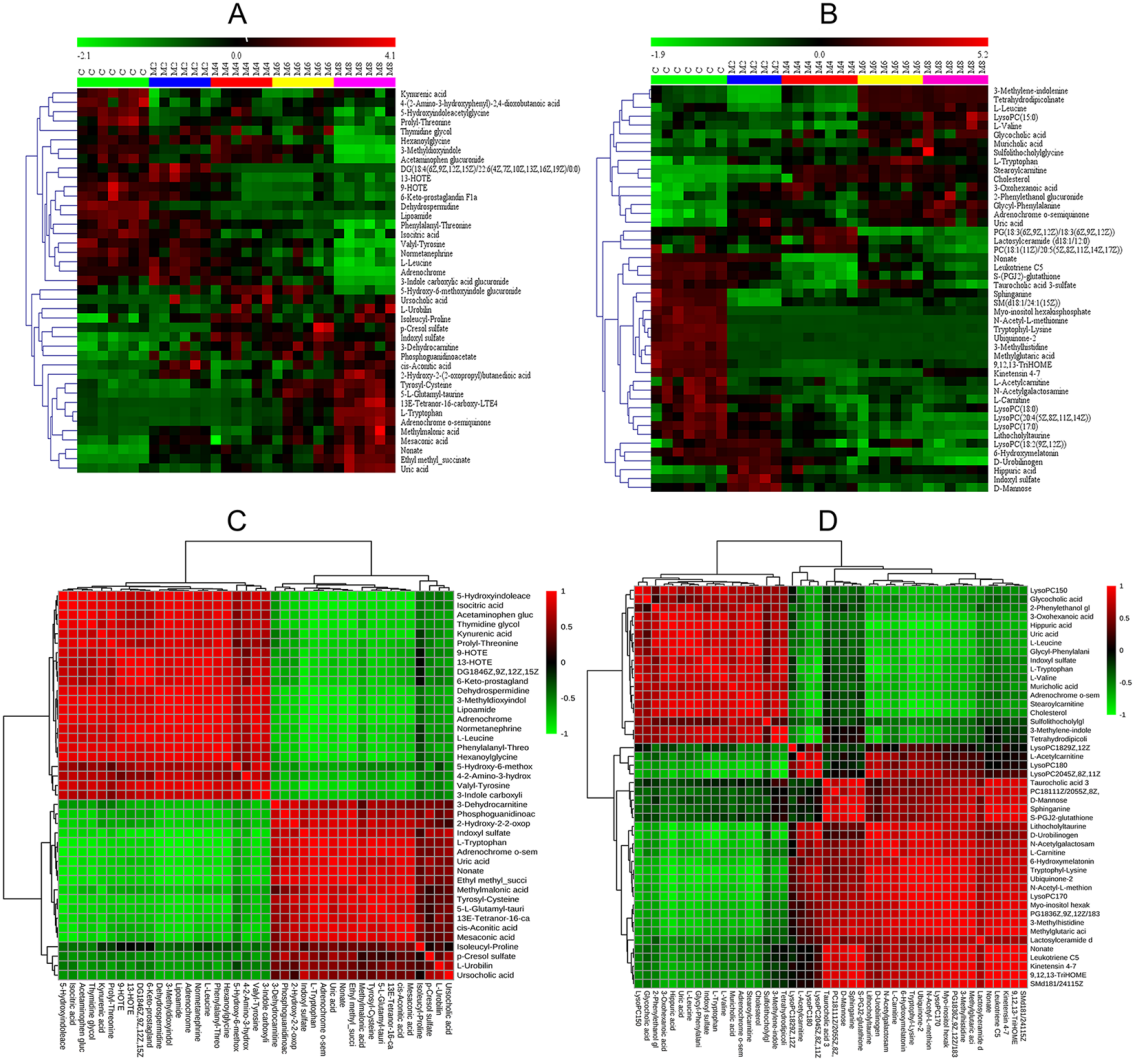
Heat-map of differential metabolite abundance levels at weeks 2, 4, and 8. (**A**) Metabolic profiles of urine samples. (**B**) Metabolic profiles of serum samples. Rows: samples; columns: metabolites. The degree of colour saturation indicates the metabolite expression value, with green: lowest; red: highest; and black: mean. Correlation matrix of differential metabolites created using Pearson’s linear correlation analysis. (**C**) Metabolic profiles of the urine samples. (**D**) Metabolic profiles of the serum samples. The colour saturation of red or blue represents the positive and negative correlation coefficients between markers, respectively.

Identified metabolites were selected according to their variable importance in projection (VIP) values of V-plots (Supplementary Fig. S2E) between the model group at week 8 and the control group. Generally, the VIP values reflect the influence of each variable, with a larger distance indicating a more important projection. Therefore, the urine and serum VIP value criteria were separately raised to narrow down the targets. Finally, a total of 41 variables in the serum were selected with VIP values greater than 3.0 and P-values (t-test) less than 0.05. Similarly, 46 variables in the urine were selected with VIP values greater than 2.0 and P-values(t-test) less than 0.05. The detailed method for compound identification has been previously reported^23^. The PLS-DA models (Fig. 3A,B) based on these differences showed that the model group could be clearly separated from the control group at week 8, and thus these metabolites could potentially be regarded as differential metabolites representing the metabolic characteristics of liver fibrosis. Meanwhile, class permutation tests were shown in Fig. 3C,D to prospectively evaluate the predictability of these PLS-DA models. Additionally, to explore the changes in these markers of liver fibrosis at different evolutionary stages, we compared the concentrations of these metabolites in the model group at other time points (weeks 2, 4, and 6). The P-values from the t-test and ANOVA were also applied to calculate the significance of each identified metabolite. The final results of the different identified metabolites are shown in Supplementary Table S2. Furthermore, the differentially abundant metabolites were visualized as a heat-map, which directly showed changes in identified metabolites in the urine and serum between the control and model groups at weeks 2, 4, 6 and 8 (Fig. 4A,B). Monitoring changes in these metabolites may predict liver fibrosis development. Correlation analysis (Fig. 4C,D) showed a good correlation between these identified metabolites.

**Figure 5:**
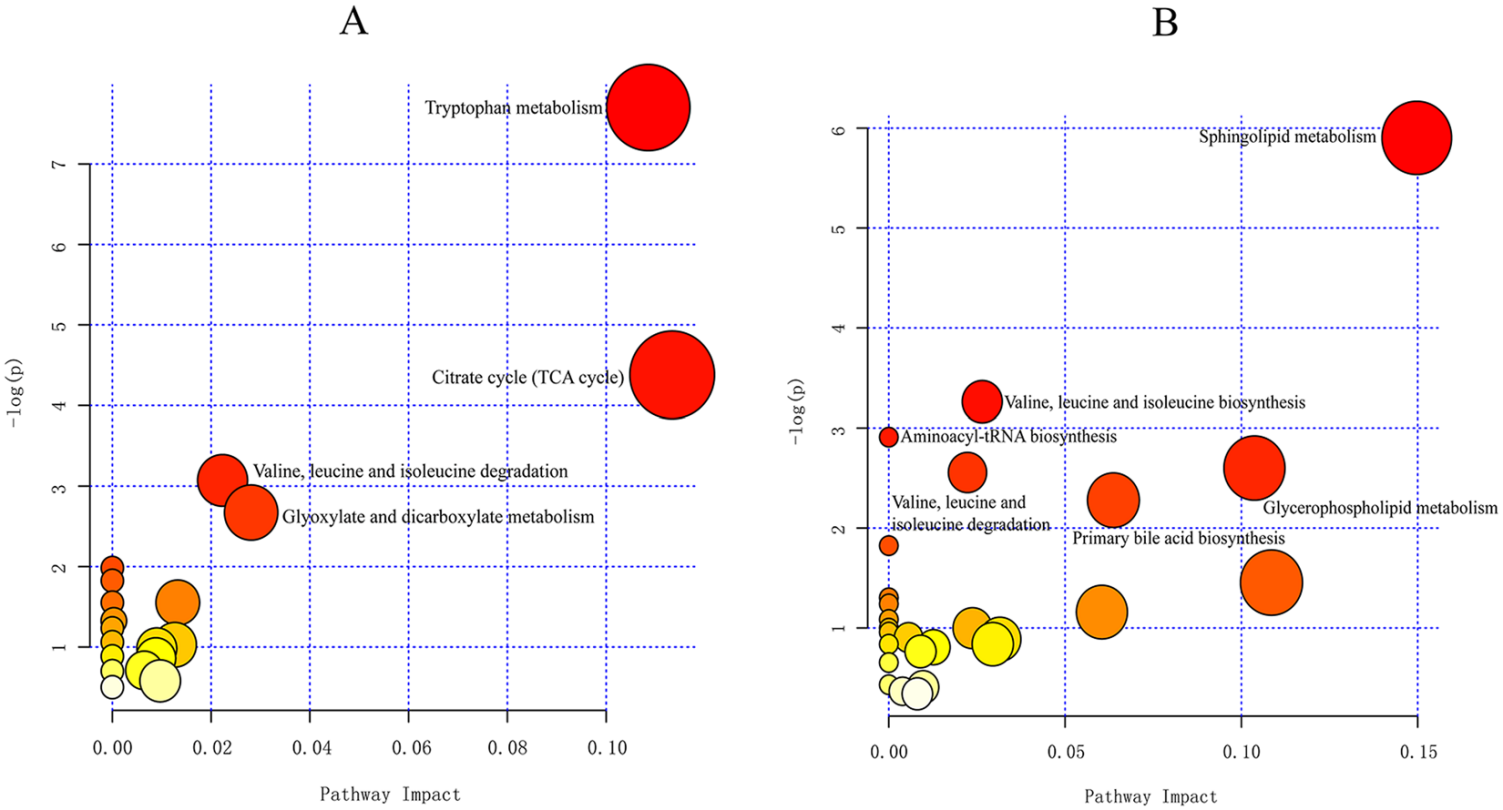
Summary of the altered metabolism pathways with MetPA, as analysed using MetaboAnalyst 3.0. (**A**) Metabolism pathway analysis of urine samples. (**B**) Metabolism pathway analysis of serum samples. The size and colour of each circle was based on pathway impact value and P-value, respectively.

To further explore the impacts of these selected metabolites and to identify possible biochemical pathways that were affected by liver fibrosis, MetPA (Metabolomics Pathway Analysis) was constructed to reveal the most relevant pathways affected by liver fibrosis. Figure 5 and Supplementary Table S3 show four metabolic pathways in urine involved in tryptophan metabolism, the citrate (TCA) cycle, valine, leucine and isoleucine degradation and glyoxylate and dicarboxylate metabolism, as well as six metabolic pathways in serum involved in sphingolipid metabolism, valine, leucine and isoleucine biosynthesis, aminoacyl-tRNA biosynthesis, glycerophospholipid metabolism, valine, leucine and isoleucine degradation and primary bile acid biosynthesis. These metabolic pathways showed marked levels of dysregulation (−Log(p) > 2) over the time-course of liver fibrosis and might facilitate liver fibrosis development. Finally, nine crucial metabolites in urine were identified from these selected metabolic pathways as follows: five metabolites (L-tryptophan, kynurenic acid, 4-(2-amino-3-hydroxyphenyl)-2,4-dioxobutanoic acid 5-hydroxyindoleacetylglycine, and 3-methyldioxyindole) are metabolic products of the tryptophan metabolic pathway; two metabolites (cis-aconitic acid and isocitric acid) belong to the TCA cycle and glyoxylate and dicarboxylate metabolic pathways; and two metabolites (L-leucine and methylmalonic acid) belonged to the valine, leucine and isoleucine degradation pathways. Additionally, ten crucial metabolites were identified in the serum, including three metabolic products (sphinganine, lactosylceramide, and sphingomyelins) of sphingolipid metabolism, three metabolic products (L-valine, L-leucine, and L-tryptophan) of aminoacyl-tRNA biosynthesis, two metabolic products (LysoPC(17:0) and PC(18:1(11Z)/20:5(5Z,8Z,11Z,14Z,17Z)) of glycerophospholipid metabolism and two metabolic products (cholesterol and glycocholic acid) of primary bile acid biosynthesis. These products might be crucial biomarkers associated with the mechanisms underlying liver fibrosis initiation and progression.

### Changes and diagnostic potential of crucial metabolites

**Figure 6:**
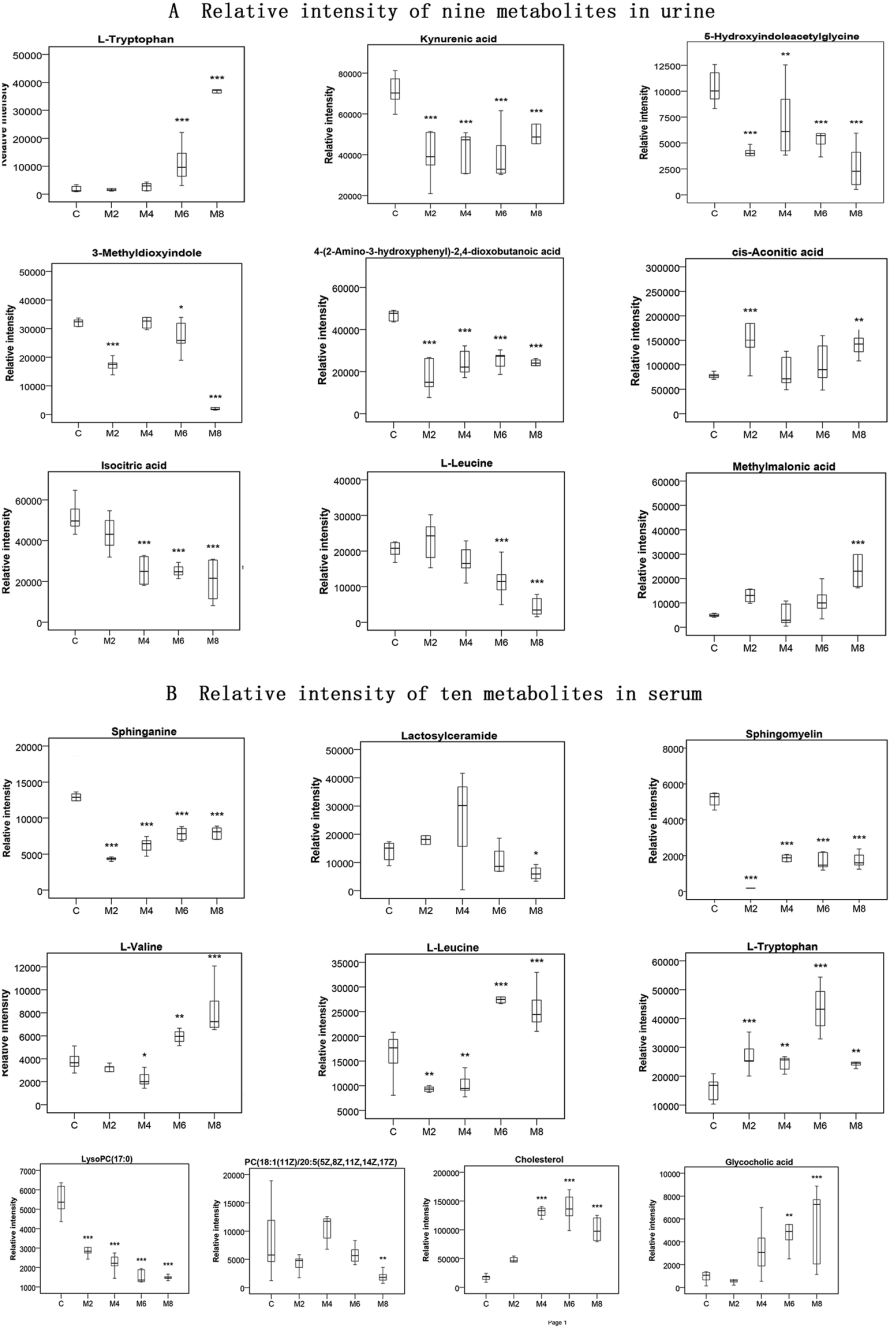
Combined box-and-whisker plots for the changes of crucial metabolites at different evolutionary stages of liver fibrosis. (**A**) Relative intensities of nine urine metabolites from weeks 2 to 8. (**B**) Relative intensities of ten serum metabolites from weeks 2 to 8. *P < 0.05, **P < 0.01, ***P < 0.001 significant differences compared to the control group.

Combined box-and-whisker plots are presented in Fig. 6 showing changes in key biomarkers of progressive liver fibrosis, from early and intermediate to advanced stages. These metabolites were significantly increased or decreased at different stages of liver fibrosis within 2 to 8 weeks. Five metabolites (kynurenic acid, 5-hydroxyindoleacetylglycine, 4-(2-amino-3-hydroxyphenyl)-2,4-dioxobutanoic acid, 3-methyldioxyindole, and cis-aconitic acid) in the urine and five metabolites (sphinganine, sphingomyelin,﻿ L-leucine, L-tryptophan, and LysoPC(17:0)) in the serum showed profound changes at week 2; as such, they could be indicative of early liver injury prior to the appearance of conventional histological abnormalities. Moreover, except for 3-methyldioxyindole and cis-aconitic acid, all of these metabolites were changed at all time points. To this end, these metabolites might be considered to be associated with the development of liver fibrosis and its early diagnosis as biomarkers. Interestingly, the alteration of cis-aconitic acid was most dramatic at the early time point and less pronounced in the advanced stage. Given this finding, we speculated that cis-aconitic acid might be identified as a potential biomarker to discriminate the early and advanced stages of liver fibrosis. In addition, three metabolites (L-tryptophan, isocitric acid, L-leucine) in urine and three metabolites (L-valine, cholesterol, glycocholic acid) in serum were increased at the later stages of liver fibrosis (week 6 and 8). Methylmalonic acid in urine and lactosylceramide, PC(18:1(11Z)/20:5(5Z,8Z,11Z,14Z,17Z)) in serum were detected at week 8 only. These variations of metabolites allowed us to obtain some important metabolic information about the mechanisms involved in the evolution of liver fibrosis.

**Figure 7:**
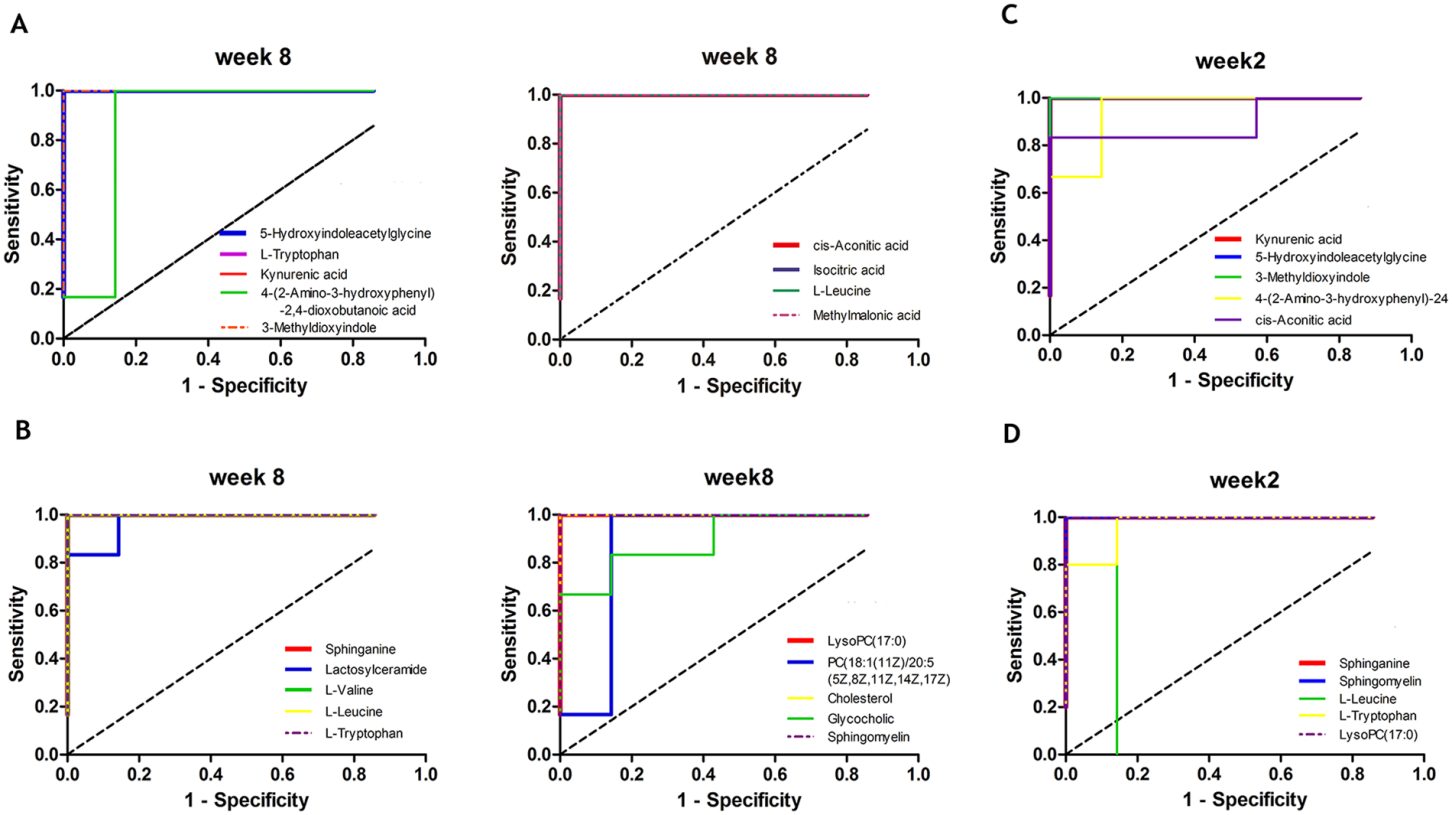
ROC curves for the diagnosis of liver fibrosis based on the potential biomarkers in urine (**A**) and serum (**B**) samples at week 8 and urine (**C**) and serum (**B**) samples at week 2. AUC, area under the curve.

Next, to further validate the significance of these metabolites as potential liver fibrosis biomarkers, receiver operating characteristic curve (ROC) analyses were performed at week 8 (Fig. 7A,B and Supplementary Table S4) with areas under the curve (AUCs), sensitivities and specificities and 95% confidence intervals (95%CIs). As presented here, all of these metabolites were identified as the top-ranked candidates that can significantly increase diagnostic performance of the metabolic markers with AUC values of more than 0.85. Among these potential candidates, ten metabolites serving as biomarkers of early liver injury were also examined by ROC analysis (Fig. 7C,D and Supplementary Table S5) at week 2. Similarly, their AUC values were also greater than 0.85. Moreover, eight of these metabolites (5-hydroxyindoleacetylglycine, 4-(2-amino-3-hydroxyphenyl)-2,4-dioxobutanoic acid, kynurenic acid, sphinganine, sphingomyelin, L-leucine, LysoPC(17:0), and L-tryptophan) were profoundly changed in the model group from week 2 to week 8 and thus could be regarded as effective biomarkers for the early detection of liver fibrosis.

## Discussion

**Figure 8:**
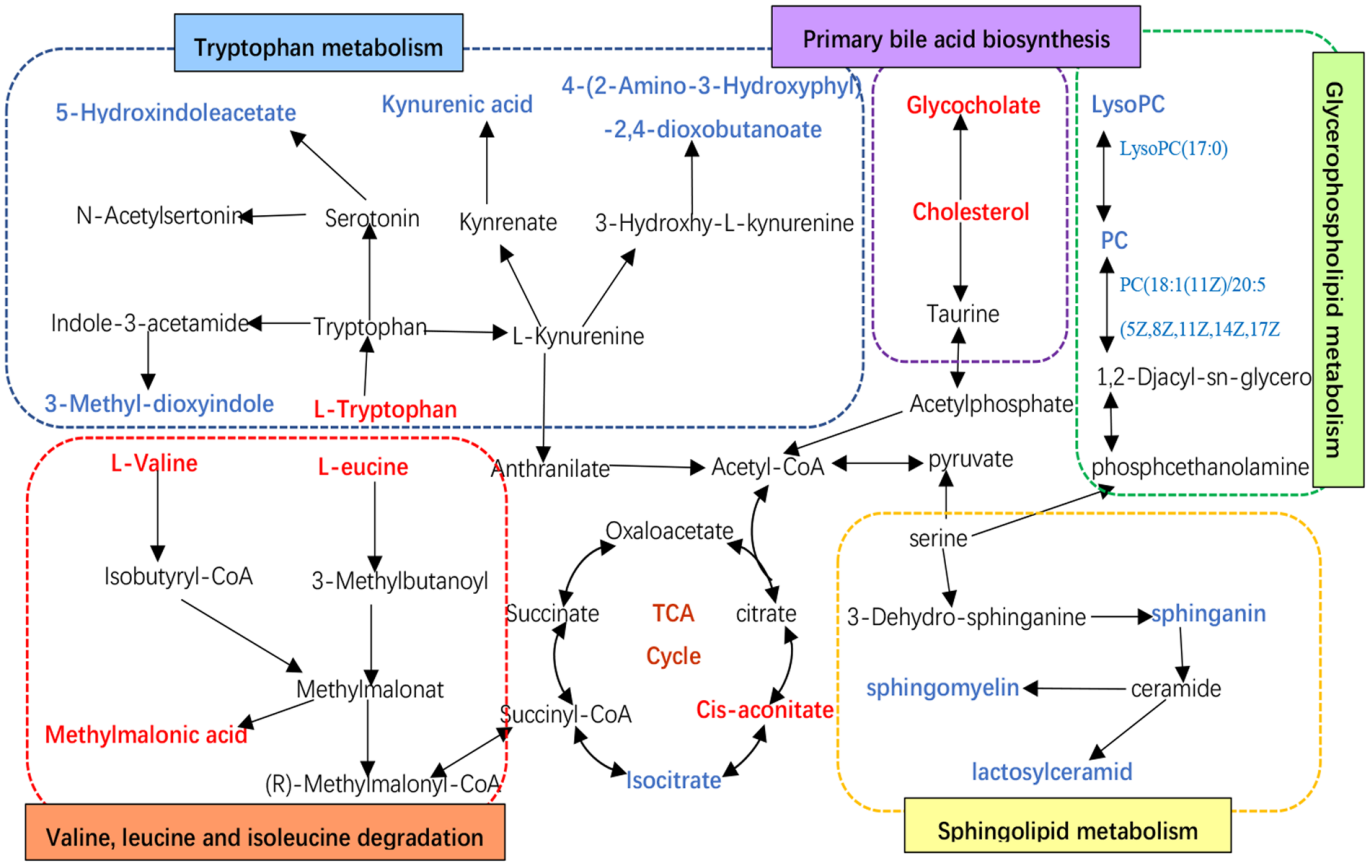
Network of the identified key biomarkers and pathways according to the KEGG pathway database. The metabolites coloured blue or red represent declining or increasing levels, respectively, in the rats with liver fibrosis at week 8.

Identification of sensitive and specific biomarkers for the early detection of hepatic fibrosis is of great importance. The metabolomics method is a useful tool for discovering diagnostic and therapeutic biomarkers by analysing global metabolic profile changes. In this study, UPLC-QTOF/HDMS-based urinary and serum metabolomics revealed changes in metabolic pathways, including amino acid metabolism (tryptophan, valine, leucine, and isoleucine metabolism), the TCA cycle, and dicarboxylate metabolism in the urine (Fig. 8) and in amino acid metabolism (valine, leucine, and isoleucine metabolism), sphingolipid metabolism, aminoacyl-tRNA biosynthesis, glycerophospholipid metabolism, and primary bile acid biosynthesis in the serum (Fig. 8). These changes occurred during liver fibrosis initiation and progression in CCl_4_-treated rats and were closely related to liver fibrosis initiation and progression. Among these metabolites, it is worth noting that urinary kynurenic acid, 4-(2-amino-3-hydroxyphenyl)-2,4-dioxobutanoic acid, 5-hydroxyindoleacetyl glycine, and serum sphinganine, sphingomyelin, LysoPC(17:0), L-leucine, and L-tryptophan changed at all time points in this study, Therefore, we hypothesize that these products are potential early biomarkers for the diagnosis of liver fibrosis and candidates for therapeutic targets.

### Changes in amino acids and their metabolites

The identified metabolites, including L-tryptophan, kynurenic acid, 5-hydroxyindoleacetyl glycine, and 3-methyldioxyindole, 4-(2-amino-3-hydroxyphenyl)-2,4-dioxobutanoic acid in urine are by-products of tryptophan metabolism. The model group showed markedly elevated urinary L-tryptophan at weeks 6 and 8 and significant decreases in four other metabolites at all time points, suggesting a disturbance of tryptophan metabolism during the onset and evolution of liver fibrosis (Supplementary Fig. S3).

L-tryptophan is an essential amino acid necessary for protein biosynthesis and is mostly metabolized via the kynurenine (Kyn) and serotonin pathways. Biosynthesis of reactive oxygen species (ROS) molecules plays a central role in the pathogenesis of liver damage and fibrosis^20, 24^. The previous study suggested that L-tryptophan exacerbated the hepatic steatosis, ROS production, liver injury and fibrosis induced by a high-fat and high-fructose diet in mice^25^. Although L-tryptophan is mostly used for protein synthesis, a part of L-tryptophan is used for serotonin and kynurenine synthesis. Tryptophan can be converted into kynurenine by indoleamine-2,3-dioxygenase and then converted to kynurenic acid (KYNA) by aminotransferase^9^. The Kyn pathway correlates with inflammation and oxidative stress (SOX), which has been postulated to be crucially involved in the pathogenesis of tissue fibrosis^26–28^. KYNA is a ligand for the orphan G protein-coupled receptor 35 (GPR35). The activation of this receptor inhibits the release of TNF-α (tumour necrosis factor) by macrophages under lipopolysaccharide(LPS)-induced inflammatory conditions. Additionally, GPR35 decreases intracellular Ca^2+^ by inhibiting its entrance. Therefore, KYNA most likely exerts an anti-inflammatory effect^29^. KYNA is also an endogenous antioxidant that can scavenge hydroxyl radicals and peroxynitrite^20^. Further, there is evidence that reduced concentrations of KYNA can be found in some types of tissue fibrosis, such as atherosclerosis and renal fibrosis^9, 29^.

In addition, we also found that the serum levels of valine and leucine were dramatically decreased during liver fibrosis onset and evolution and conversely increased in the advanced stage (Supplementary Fig. S4). Interestingly, urinary levels of L-leucine were decreased by 6 and 8 weeks. L-leucine is a branched amino acid involved in liver protein synthesis, and its deprivation induces liver steatosis in Gcn2 knock-out mice^30^. Nakanishi *et al*. reported that L-valine could improve liver disease by ameliorating the thrombopoietin production of hepatocytes and hepatic fibrosis by promoting recovery from liver injury^31^. Additionally, the ratios of branched-chain amino acids (BCAAs, valine, leucine and isoleucine) to aromatic amino acids (tyrosine, phenylalanine and tryptophan) were lower in HBV (hepatitis B virus), LC (liver cirrhosis) and HCC (hepatocellular carcinoma), indicating that enhanced valine and leucine catabolism and reduced tryptophan breakdown are associated with liver diseases^32^. Consistently, our data show a marked decrease in L-valine and L-leucine at an early stage in the CCl_4_-treated group that is indicative of liver injury and may contribute to the progression of liver fibrosis. However, more research is needed to confirm whether the serum levels of valine and leucine are increased in the advanced stage of liver fibrosis and to investigate the mechanisms underlying their beneficial effects.

### Changes in TCA cycle

The TCA cycle is the final common oxidative pathway for fats, carbohydrates and amino acids and is the most important central pathway connecting almost all individual metabolic pathways^33^; additionally, the TCA cycle has a close relationship with some liver diseases. Satapati S. *et al*. reported that lipid accumulation and a loss of insulin action resulted in the elevation of the oxidative and anaplerotic pathways of the hepatic TCA cycle in mice, which progressed to insulin resistance and a fatty liver due to a high-fat diet^34^. However, liver fibrosis occurred due to the on-going liver damage induced by oxidative stress, and the TCA cycle was slowed by cellular regulation to reduce the natural production of ROS; this effect has been related to liver diseases caused by various mechanisms^20^. Similarly, in our study, the model group showed significantly decreased urinary isocitric acid (TCA cycle intermediates) at weeks 4, 6 and 8 and significantly accumulated cis-aconitic acid (TCA cycle intermediates) at weeks 2 and 8 compared with the normal group (Supplementary Fig. S5), which suggested the participation of an altered TCA cycle during the entire time-course of CCl_4_-treated liver fibrosis in rats.

### Changes in sphingolipid metabolism

Sphingolipid metabolism has a crucial role in liver fibrosis progression, which has been demonstrated in earlier studies^17, 35, 36^. The liver fibrosis rats employed in our study exhibited significantly decreased serum sphinganine, lactosylceramide and sphingomyelin levels (Supplementary Fig. S6).

Altered sphingolipid metabolism is potentially related to the inflammatory response and ceramide increase^26^. Ceramides couple with sphingomyelins, free sphingoid bases and their phosphates, and complex glycosphingolipids compose the sphingolipids, which are regarded as a highly diverse class of lipids^35^. These lipids participate in mixed signal processes, including proliferation, differentiation and apoptosis^36, 37^, and thus potentially affect the pathogenesis of various diseases, especially those involving tissue fibrosis^38^. In particular, our data indicate that changes occur in the sphinganine and sphingomyelin levels induced by the dysregulation of sphingolipid metabolism at all stages in the liver fibrosis group and that these metabolites may represent potential markers for use in the early non-invasive prediction of liver fibrosis.

### Changes in glycerophospholipid metabolism

Glycerophospholipids (GPs), as storage deposits for lipid mediators, function as integral membrane proteins, transporters, receptors and ion channels^39^. CCl_4_-treated rats can be subjected to perturbations of lipid metabolism to induce liver fibrosis^17^. Lysophospholipids are metabolites of GP metabolism and can be transiently produced during the remodelling of GPs^40^. Previous reports have indicated that LysoPCs played an essential role in the progression of liver injury. In the early stage of liver injury, LysoPC deficiency reflected a rapid membrane phosphatidylcholine turnover^41^. Phosphatidylcholine (PC), as an important supporting nutrient for the liver, exerted an obvious protective effect against liver damage from viruses, pharmaceuticals, alcoholism and other toxic influences^42^. PC was also effective against fibrosis and cirrhosis in the baboon^39^. Consistently, in this study, the LysoPC (17:0) and PC (18:1(11Z)/20:5(5Z,8Z,11Z,14Z,17Z) levels were significantly attenuated in the serum of the CCl_4_-treated group, indicating that GP metabolism was down-regulated in the process of liver fibrosis.

### Changes in primary bile acid biosynthesis

Bile acids are normally maintained at a balance of free and conjugated forms in peripheral circulation. When hepatic or intestinal diseases occur, the balance will be damaged, especially in the synthesis, reabsorption and excretion of bile acids, which can lead to elevated levels of total bile acid^43^. Previous studies have reported liver injury caused by liver diseases, such as cirrhosis and gallbladder disease, results in a decrease in the hepatic clearance of bile acids and eventually an increase in the levels of bile acids in the serum^44^. Furthermore, bile acid metabolism was also reported to be involved in inhibiting the increase of excretion in liver fibrosis rats^43^. Recent studies have demonstrated that bile acids may facilitate liver injury and ultimately promote cirrhosis or liver failure by modulating bile acid-induced apoptosis and necrosis of hepatocytes^41^. As noted above, bile acids are considered to be a hallmark of liver injury. In our study, cholesterol and glycocholic acid, which are regarded as conjugate bile acids, were dramatically increased during the intermediate and advanced stages in the CCl_4_-treated group. These findings indicated the participation of altered serum lipid metabolism in the pathogenesis of liver fibrosis.

In summary, we performed urinary and serum metabolomics investigations in rats with liver fibrosis, provided a holistic understanding of disease progression, and laied the foundation for future development of liver fibrosis-specific biomarkers for early clinical disease prediction and diagnosis. The present findings also revealed significant fibrotic pathways and dynamic changes in different stages of liver fibrosis. Perturbations of tryptophan, valine, leucine, isoleucine, the TCA cycle, sphingolipid metabolism and GP metabolisms occurred from the onset of liver fibrosis. Additionally, profound changes in valine and bile acid biosynthesis metabolites were observed in the intermediate and advanced stages. However, the potential interplay and correlation of these key metabolites and their underlying mechanisms in the pathogenesis of liver fibrosis are unclear and require further investigation. Moreover, due to some technical limitations in this study, more samples and multi-analytical techniques are needed to validate the diagnostic performance of these findings and to develop their diagnostic assays. Since these metabolomic abnormalities were detected only in the CCl_4_-treated liver fibrosis model, more metabolomic studies of liver fibrosis in animals and humans are required to associate these results with a clinical diagnosis. Furthermore, a combinatory metabolite panel is also expected to reveal the comprehensive mechanism of liver fibrosis at the molecular level.

## Materials and Methods

### Chemicals and reagents

CCl_4_ was purchased from Tianjin Beifang Chemical Company (Tianjin, China). Olive oil was purchased from Shanghai Chemical Reagent Company (Shanghai, China). Acetonitrile (HPLC grade) was purchased from Thermo Fisher Scientific (USA). Deionized water was produced using a Milli-Q ultrapure water system (Millipore, Billerica, USA). Formic acid (HPLC grade) was purchased from Dikma Technologies (USA). Leucine enkephalin was purchased from Sigma-Aldrich (USA). All other reagents were of HPLC grade.

### Animals

Male Sprague-Dawley (SD) rats (1 year old, 180–200 g) were obtained from the Drug Safety Evaluation Center (Heilongjiang University of Chinese Medicine). The animals were housed at a controlled temperature of 26–28 °C, humidity 55% ± 5% and with 12-h light and 12-h dark cycles. Water and food were made available to the rats ad libitum. After a weeklong acclimation period, rats were randomly divided into control (n = 7) and model (n = 24) groups. Hepatic fibrosis was induced by subcutaneous injection of CCl_4_ (1 mL/kg 40% CCl_4_, diluted in olive oil) twice a week for eight weeks. The control group was exposed to olive oil at the same volume as the other group but without CCl_4_ for eight weeks. The animal experiment was performed according to the Guidelines for Animal Experimentation of the Heilongjiang University of Chinese Medicine, and the animal study was approved by the Animal Ethics Committee.

### Sample collection and preparation

All operations were performed under sterile conditions. The overnight urine samples (12 h) were collected in metabolism cages from the control (n = 7) and model groups (n = 6) every week, and the blood was collected from the orbital vein at weeks 2, 4, 6 and 8. Urine samples were centrifuged at 12,000 g for 10 min, and the supernatant was flash-frozen in liquid nitrogen and stored at −80 °C until analysis. Blood was centrifuged at 900 × g for 10 min, and the supernatants were transferred into Eppendorf tubes and stored at −80 °C for UHPLC/MS analysis. Six rats from the model group were sacrificed at weeks 2, 4, 6 and 8, and rats from the control group were sacrificed at week 8. Liver tissues were washed with saline buffer and fixed in 4% buffered paraformaldehyde until use for pathological observation.

### Histological assessment

Paraformaldehyde-fixed liver tissues were processed, and 4-µm-thick slices were stained with H&E. The injury score of fibrosis was graded according to the description by Ishak^45^ in ten randomly selected non-overlapping fields per rat. Moreover, liver sections were subjected to Masson’s trichrome stain. To quantify the liver fibrosis, the blue pixel contents of the images were photographed using the same microscope and magnification times. Five different areas of the tissue slide from each rat were selected to detect the values of the integral optical density and the total area, and the expression intensity was calculated as the percentage of the integral optical density to the total area, which was analysed using Image-Pro Plus 6.0.

### UPLC-QTOF-MS assay

All the urine and serum samples were thawed in an ice water bath and vortex-mixed before analysis. Urine sample preparation and sample analysis by UPLC-Q-TOF were described in detail in the Supplementary Information.

### Multivariate data analysis

The raw data were analysed using MassLynx V4.1 and MarkerLynx software (Waters Corp., Milford, USA). The multivariate data matrix was analysed using EZinfo software (Waters Corp., Milford, USA). An unsupervised PLS-DA was performed to visualize general clustering and trends. OPLS-DA was utilized to validate the PCA model. The parameters (R^2^X, R^2^Y, and Q^2^) were calculated to explain the goodness of fit and the predictive capability of the model. R^2^Y and Q^2^ values close to 1.0 represent an excellent model. Differential metabolites were extracted from the combined V-plot constructed from the loading plots of the OPLS-DA between the control and model groups at week 8, and the differences were determined using the VIP values and P-values from the two-tailed Student’s t-test. Classical one-way analysis of variance (ANOVA) performed in IBM SPSS Statistics 23 was used to further select and judge the statistical significance of the results. The heat-map implemented by the Multi Experiment Viewer, which is commonly used for unsupervised clustering, was constructed based on these differential metabolites. Additionally, ROC analysis was performed to select candidate biomarkers, and the classification performance (1-specificity and sensitivity with the highest accuracy) was assessed according to the AUC values of the ROC curves.

### Metabolite identification and pathway analysis

Exact molecular mass data from redundant m/z peaks were used to help confirm the metabolite molecular mass. The mass tolerance between the measured m/z values and the exact mass of the components of interest was set to within 5 mDa. The MassFragment™ application manager (Waters MassLynx V 4.1) was used to facilitate the MS/MS fragment ion analysis. The metabolic pathway analysis of potential biomarkers of liver fibrosis was performed with Metabolomics Pathway Analysis (MetPA) using MetaboAnalyst software 3.0 (http://www.metaboanalyst.ca/Metabo-Analyst/) to identify the top altered pathways analysis^46^. Network analysis of the identified biomarkers showed their interrelationships based on the Kyoto Encyclopaedia of Genes and Genomes (KEGG) pathway database (http://www.genome.jp/kegg/)^47^.

## Electronic supplementary material

Supplementary Information
