# Supplementary material for: Identification of key metabolic changes during liver fibrosis progression in rats using a urine and serum metabolomics approach
Source: Sci Rep. 2017 Sep 12;7:11433. doi: 10.1038/s41598-017-11759-z (PMC5595818; doi:10.1038/s41598-017-11759-z)
Supplement: Supplementary file 1 — Supplementary Information [file 41598_2017_11759_MOESM1_ESM.pdf]

Supplementary information to:

Identification of key metabolic changes during liver fibrosis progression in rats using a urine and serum metabolomics approach

Hong Chang[1,3], Hong-yu Meng[1], Shu-min Liu[1,2*], Yu Wang[1], Xiao-xu Yang[1], Fang Lu[1] & Hong-yu Wang[1]

[1] Chinese Medicine Toxicological Laboratory, Heilongjiang University of Chinese Medicine, Harbin, P.R. China

[2] Drug Safety Evaluation Center, Heilongjiang University of Chinese Medicine, Harbin, P.R. China

[3] School of Pharmacy, Baotou Medical College, Inner Mongolia, Baotou, P.R. China

Correspondence
Shu-min Liu
Drug Safety Evaluation Center
Heilongjiang University of
Chinese Medicine
He Ping Road 24
Harbin 150040
P. R. China
Phone: + 86045182193278
keji-liu@163.com

UHPLC conditions

Waters Acquity™UHPLC (consisting of a vacuum degasser, autosampler, binary pump, photodiode array detector, and oven) was equipped with an ACQUITY UPLC® BEH C18 column (2.1mm×50mm, i. d. 1.7 μm, Waters Corp). The analytical column was maintained at a temperature of 40°C and the mobile phase was composed of acetonitrile (A) and water (B), each containing 0.1% formic acid. The gradient for the urine sample was as follows: 0-8min, 1-40%A; 8-10min, 40-98%A; 10-13min, 98-100%A. The gradient for serum sample was used: 2–100% A for 15 min. The injection volume was 2 μL with a flow rate of 0.4 mL/min. The eluent was introduced to the mass spectrometer directly.

MS conditions

MS analysis was performed on a Q-TOF analyzer in the SYNAPTHDMS system (Waters Corporation) in the positive-ion (ESI^+) and negative-ion (ESI^-) mode, using the following parameters: capillary voltage, 1300V(ESI^+), 1500V(ESI^-); sample cone voltage, 60 V(ESI^+), 770V(ESI^-); source temperature, 110°C; desolvation temperature, 350°C; desolvation gas flow, 750 L/h; cone gas flow, 20 L/h. MS data were collected in the full scan mode from m/z 100–1500. All the data were acquired using an independent reference lock mass via the LockSpray™interface to ensure accuracy and reproducibility during the MS analysis. Leucine encephalin was used as the reference ion ($[M+H]^+$ = 556.2771) and $[M-H]^-$ = 554.2615) at a concentration of 1 ng/mL under a flow rate of 30 μL/min. The data were collected in the centroid mode, and the LockSpray frequency was set at 15 s and was averaged over five scans for correction.

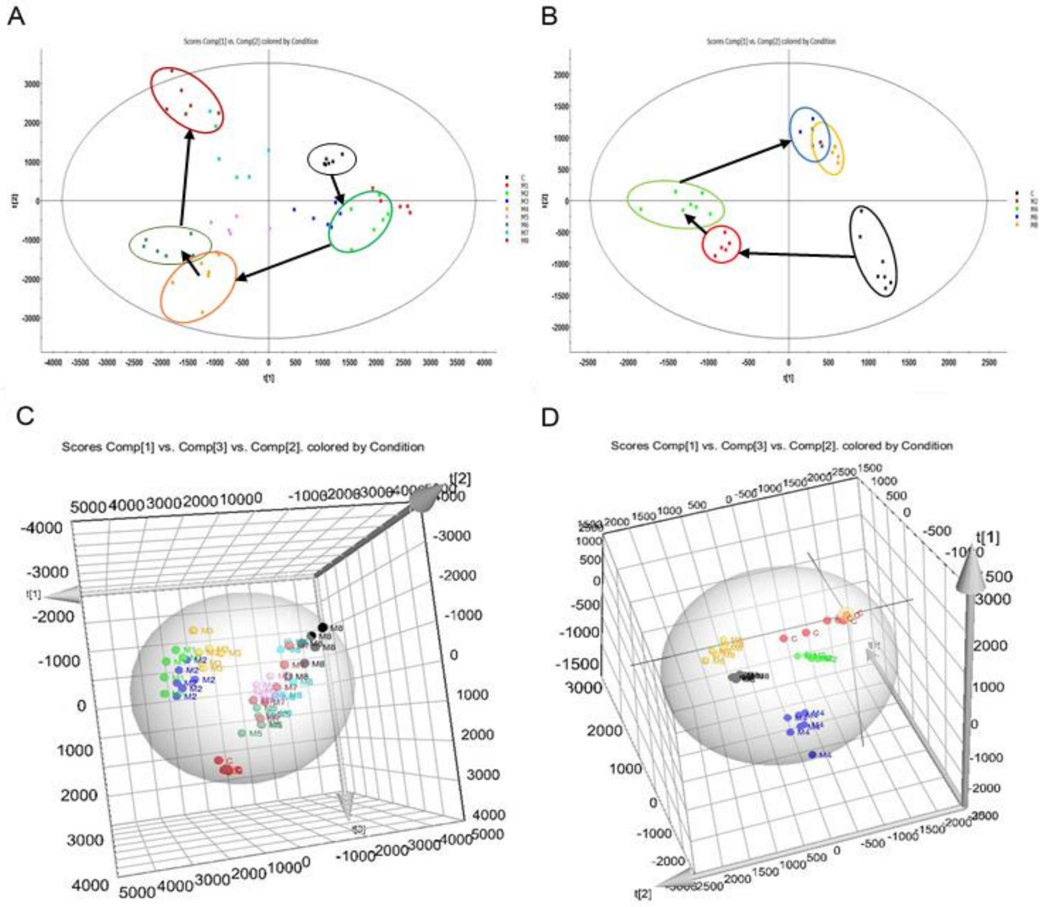

Figure S1. Trajectory analysis of PLS-DA and 3D PLS-DA Score plots for the liver fibrosis. (A, C) the urine samples in negative mode (R^2Y=53%, Q^2=44%). (B, D) The serum samples in negative mode (R^2Y=97%, Q^2=95%).

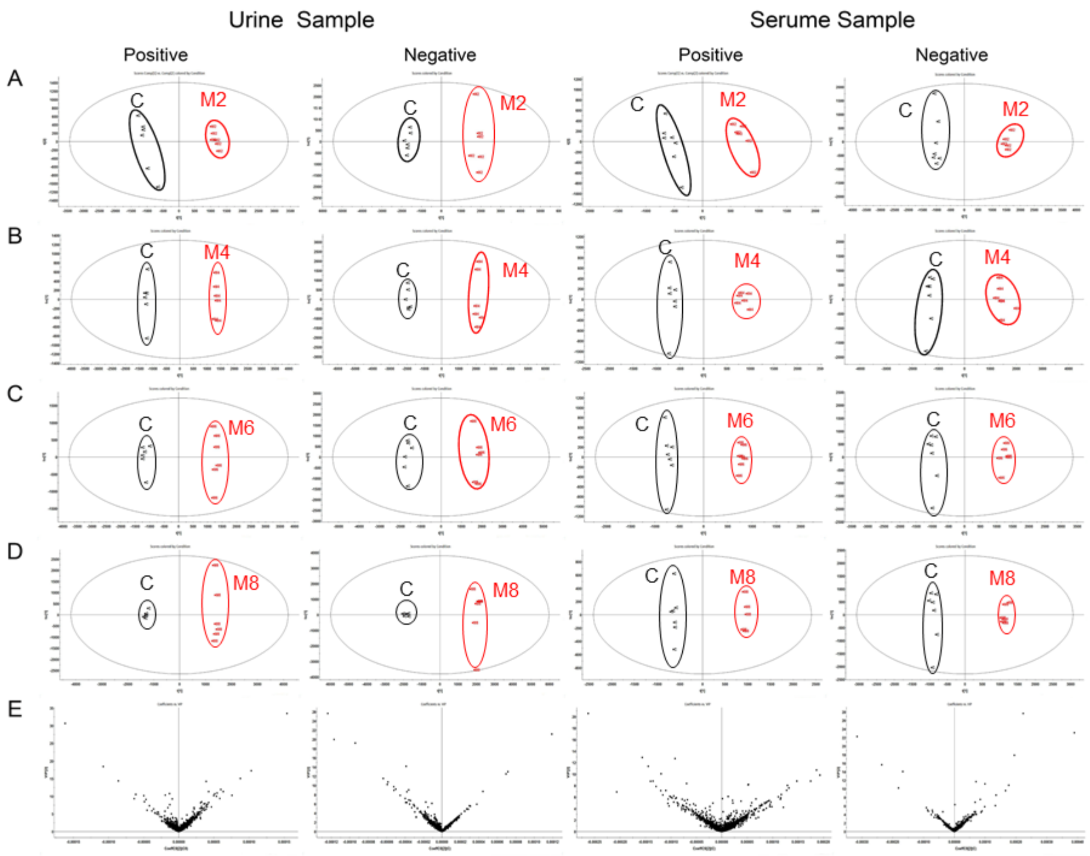

Figure S2. The screening of the metabolite markers. OPLS-DA score plots of urine and serum samples analysed in positive and negative modes to compare liver metabolite profiles between the control and model groups at weeks 2(A), 4(B), 6(C), and 8(D). (E) VIP score plots constructed from OPLS-DA models of urine and serum samples at week 8.

Table S1 Cross-validated data of OPLS-DA models in liver fibrosis model groups compared to control group.

**Model**	**Positive ion mode**			**Negative ion mode**		
	**R^2X**	**R^2Y**	**Q^2**	**R^2X**	**R^2Y**	**Q^2**
Urine						
C vs M2 (week 2)	0.49	0.99	0.96	0.64	0.99	0.97
C vs M4 (week 4)	0.58	0.99	0.98	0.71	0.99	0.97
C vs M6 (week 6)	0.59	0.99	0.98	0.68	0.99	0.96
C vs M8 (week 8)	0.74	0.99	0.97	0.66	0.99	0.97
Serum						
C vs M2 (week 2)	0.69	0.99	0.96	0.85	0.98	0.97
C vs M4 (week 4)	0.70	0.98	0.97	0.85	0.98	0.97
C vs M6 (week 6)	0.69	0.99	0.96	0.80	0.99	0.98
C vs M8 (week 8)	0.69	0.99	0.99	0.82	0.98	0.99

Table S2. Differential metabolites from different groups at weeks 2, 4, 6 and 8.

No.	Metabolite	m/z	RT (min)	HMDB	2weeks model vs control			4weeks model vs control			6weeks model vs control			8weeks model vs control			
					FC[a]	P^b	P^c	FC[a]	P^b	P^c	FC[a]	P^b	P^c	FC[a]	P^b	P^c	VIP[d]
urine																	
1	Valyl-Tyrosine	162.06	3.69	HMDB29139	-0.54	4.42E-02	2.29E-02	-1.11	2.82E-04	5.11E-04	-1.21	2.34E-04	4.88E-04	-3.02	3.70E-10	4.85E-06	9.05
2	Ursocholic acid	385.14	3.70	HMDB00917	2.67	P^b >0.05	P^c >0.05	3.64	1.35E-02	1.89E-02	2.62	2.20E-01	P^c >0.05	2.94	P^b >0.05	8.99E-03	3.50
3	Uric acid	160.04	3.69	HMDB00289	0.43	P^b >0.05	P^c >0.05	1.18	2.65E-02	4.18E-02	1.15	1.19E-02	6.75E-03	3.01	P^b >0.05	1.34E-05	3.91
4	Tyrosyl-Cysteine	220.12	2.02	HMDB29102	-0.01	P^b >0.05	P^c >0.05	0.02	P^b >0.05	P^c >0.05	1.5	1.72E-05	3.02E-04	2.26	9.18E-08	3.63E-05	13.69
5	Thymidine glycol	162.08	1.00	HMDB42036	-0.3	P^b >0.05	3.15E-02	-0.65	P^b >0.05	1.56E-04	-0.65	4.92E-01	4.81E-04	-0.78	1.78E-03	4.74E-06	3.13
6	Prolyl-Threonine	174.11	3.79	HMDB29027	-1.21	2.69E-05	1.32E-03	-1.08	1.00E-03	1.91E-03	-1.51	3.28E-05	3.45E-04	-1.58	8.25E-07	3.19E-04	10.13
7	Phosphoguanidinoacetate	453.28	7.96	HMDB03705	0.33	2.84E-02	P^c >0.05	0.09	4.57E-02	P^c >0.05	0.3	1.07E-03	P^c >0.05	0.78	1.86E-04	2.10E-04	4.84
8	Phenylalanyl-Threonine	328.10	2.82	HMDB29005	-0.4	P^b >0.05	2.66E-02	-0.8	2.65E-03	6.87E-05	-1.1	3.69E-05	1.97E-05	-1.6	P^b >0.05	1.10E-06	4.91
9	p-Cresol sulfate	173.01	1.01	HMDB11635	-1.85	P^b >0.05	2.38E-06	0.59	P^b >0.05	2.17E-03	1.3	2.29E-03	4.94E-02	0.89	P^b >0.05	2.67E-02	6.78
10	Normetanephrine	373.27	7.95	HMDB00819	-0.52	P^b >0.05	1.22E-02	-1.28	2.73E-04	3.98E-05	-1.54	4.40E-05	1.45E-04	-2.42	4.32E-08	2.07E-06	8.96
11	Nonate	187.10	5.37	HMDB11717	1.06	7.16E-05	2.31E-06	0.83	9.41E-05	7.57E-04	0.78	1.97E-05	1.27E-04	1.87	1.18E-10	5.25E-07	7.14
12	Methylmalonic acid	233.01	2.20	HMDB00202	1.17	P^b >0.05	1.04E-04	-0.48	P^b >0.05	P^c >0.05	0.63	1.68E-01	P^c >0.05	2.28	9.10E-06	1.75E-03	3.71
13	Mesaconic acid	189.04	1.10	HMDB00749	0.48	1.80E-03	3.80E-03	-0.21	P^b >0.05	P^c >0.05	0.08	6.89E-03	P^c >0.05	0.89	1.50E-06	1.15E-04	3.65
14	L-Urobilin	303.07	1.78	HMDB04159	-1.32	P^b >0.05	1.97E-02	0.81	P^b >0.05	P^c >0.05	-0.79	8.40E-01	P^c >0.05	1.51	5.57E-03	2.42E-02	4.73
15	L-Tryptophan	221.09	3.20	HMDB00929	-0.35	P^b >0.05	P^c >0.05	0.08	P^b >0.05	P^c >0.05	1.95	5.63E-05	1.40E-02	4.11	1.44E-16	3.21E-10	8.22
16	L-Leucine	389.27	8.81	HMDB00687	-0.12	P^b >0.05	P^c >0.05	-0.76	P^b >0.05	1.82E-03	-1.37	4.30E-04	1.58E-04	-2.57	1.34E-07	1.67E-06	5.58
17	Lipoamide	295.23	8.23	HMDB00962	-0.91	9.20E-11	1.47E-05	-2.37	3.42E-17	1.78E-10	-2.61	1.72E-17	8.94E-11	-2.39	7.89E-18	1.68E-10	4.81
18	Kynurenic acid	268.11	1.40	HMDB00715	-1.09	5.04E-06	4.23E-06	-1.18	2.28E-05	7.86E-07	-1.47	3.95E-06	1.99E-08	-0.78	1.78E-04	5.76E-05	6.07
19	Isoleucyl-Proline	334.11	1.51	HMDB28915	-0.04	P^b >0.05	P^c >0.05	-0.12	1.73E-04	P^c >0.05	-0.36	7.60E-02	2.38E-02	0.22	4.95E-04	6.08E-06	4.61
20	Isocitric acid	259.09	0.72	HMDB00193	-0.49	5.12E-02	2.74E-03	-1.48	7.60E-07	6.75E-07	-1.58	6.90E-07	3.65E-07	-1.51	4.93E-08	1.47E-06	7.34

21	Indoxyl sulfate	313.24	9.72	HMDB00682	0.31	P^b >0.05	P^c >0.05	0.95	2.60E-06	1.06E-04	0.88	9.75E-07	2.40E-05	0.89	3.52E-04	3.82E-07	19.40
22	Hexanoylglycine	661.49	8.24	HMDB00701	-0.19	P^b >0.05	P^c >0.05	-0.16	P^b >0.05	P^c >0.05	-1.65	1.63E-03	7.35E-05	-3.66	2.56E-06	1.60E-06	3.37
23	Ethyl methyl_succinate	159.07	3.24	HMDB59893	0.85	2.11E-04	3.68E-05	0.47	1.95E-03	5.33E-03	0.73	3.87E-06	3.57E-04	1.92	1.00E-12	4.84E-07	4.25
24	DG(18:4(6Z,9Z,12Z,15Z)/22:6(4Z,7Z,10Z,13Z,16Z,19Z)/0:0)	240.10	3.20	HMDB07353	1.39	5.96E-08	9.31E-04	-3.65	3.76E-03	2.68E-04	-5.45	1.99E-03	1.40E-04	-5.87	1.66E-03	1.32E-04	6.24
25	Dehydrospermidine	188.05	0.53	HMDB12925	-2.04	1.90E-19	1.36E-09	-2.7	1.58E-20	6.80E-11	-2.45	1.10E-19	1.61E-10	-1.96	2.52E-19	8.86E-10	3.81
26	cis-Aconitic acid	451.27	8.86	HMDB00072	0.8	4.83E-04	3.45E-03	-0.34	P^b >0.05	P^c >0.05	-0.18	2.95E-01	P^c >0.05	0.69	5.07E-03	8.12E-05	6.81
27	Adrenochrome o-semiquinone	205.10	2.49	HMDB12883	-1.38	P^b >0.05	7.65E-03	-0.37	P^b >0.05	P^c >0.05	1.37	6.28E-04	1.49E-02	3.53	1.97E-15	1.58E-08	10.41
28	Adrenochrome	147.03	0.88	HMDB12884	0.07	2.18E-02	P^c >0.05	-0.86	1.23E-02	8.77E-06	-1.28	2.22E-04	1.93E-07	-4.73	1.34E-10	3.48E-15	21.14
29	Acetaminophen glucuronide	267.13	4.89	HMDB10316	-0.96	4.04E-03	4.17E-03	-0.49	P^b >0.05	P^c >0.05	-1.06	1.30E-02	7.20E-03	-4.44	4.29E-08	1.31E-05	7.51
30	9-HOTE	255.07	6.03	HMDB10224	-0.97	5.11E-04	1.16E-02	-2.48	9.39E-08	2.85E-04	-2.54	1.28E-07	2.28E-04	-2.64	2.21E-08	2.09E-04	3.00
31	6-Keto-prostaglandin F1a	164.07	4.14	HMDB02886	-0.65	P^b >0.05	P^c >0.05	-1.85	2.88E-05	8.65E-04	-2.39	4.40E-06	2.34E-04	-2.62	4.28E-07	1.82E-04	3.71
32	5-L-Glutamyl-taurine	263.14	3.48	HMDB04195	-1.21	P^b >0.05	P^c >0.05	0.43	P^b >0.05	P^c >0.05	2.54	9.14E-07	1.49E-04	3.16	2.46E-08	6.43E-05	17.63
33	5-Hydroxyindoleacetylglycine	166.09	1.81	HMDB04185	-1.64	5.31E-06	2.87E-07	-1.03	5.95E-03	3.28E-04	-1.44	3.55E-04	7.18E-07	-2.17	3.33E-07	1.67E-06	3.52
34	5-Hydroxy-6-methoxyindole glucuronide	183.19	10.57	HMDB10363	0.22	P^b >0.05	P^c >0.05	1.05	1.72E-03	3.39E-02	0.77	1.01E-02	P^c >0.05	-2.64	P^b >0.05	7.37E-03	8.47
35	4-(2-Amino-3-hydroxyphenyl)-2,4-dioxobutanoic acid	206.08	5.14	HMDB04083	-1.63	1.86E-06	4.06E-04	-1.38	7.32E-05	5.52E-04	-1.41	1.99E-04	3.75E-04	-1.16	5.31E-05	1.00E-03	3.99
36	3-Methyldioxyindole	381.08	0.68	HMDB04186	-1.12	9.30E-08	2.06E-07	-0.38	P^b >0.05	1.55E-03	-0.81	1.60E-02	1.41E-05	-4.04	4.26E-14	5.87E-11	7.57
37	3-Indole carboxylic acid glucuronide	171.07	3.94	HMDB13189	0.16	P^b >0.05	P^c >0.05	-0.59	P^b >0.05	P^c >0.05	-0.97	2.92E-01	4.96E-02	-5.08	2.08E-04	3.82E-04	4.69
38	3-Dehydrocarnitine	173.08	4.42	HMDB12154	0.45	4.55E-02	P^c >0.05	0.47	2.24E-03	1.97E-02	0.59	1.26E-04	1.42E-02	0.87	5.44E-04	2.82E-03	4.94
39	2-Hydroxy-2-(2-oxopropyl)butanedioic acid	359.05	0.85	HMDB59927	1.27	4.79E-04	3.15E-03	0.32	P^b >0.05	P^c >0.05	-0.03	3.30E-01	P^c >0.05	1.83	4.48E-07	4.79E-04	3.11
40	13-HOTE	217.12	1.37	HMDB10203	0.09	P^b >0.05	P^c >0.05	-1.4	5.87E-03	1.23E-03	-1.97	8.42E-04	8.06E-05	-1.8	2.66E-04	1.59E-04	3.30
41	13E-Tetranor-16-carboxy-LTE4	215.02	0.92	HMDB12575	-1.13	P^b >0.05	4.02E-02	0.52	P^b >0.05	P^c >0.05	2.22	2.47E-02	2.82E-03	4.25	1.01E-08	1.08E-04	4.74
serum																	
1	Uric acid	167.02	0.91	HMDB00289	1.79	5.94E-03	2.45E-06	1.81	5.58E-03	9.00E-06	1.84	2.90E-04	6.23E-05	2.10	1.10E-05	7.43E-05	3.33

2	Ubiquinone-2	301.19	10.79	HMDB06709	-3.07	1.46E-20	5.16E-06	-3.07	4.37E-20	5.16E-06	-3.75	2.26E-20	5.20E-06	-3.69	2.46E-20	5.12E-06	3.03
3	Tryptophyl-Lysine	315.2	12.22	HMDB29088	-6.21	P^b >0.05	8.56E-06	-6.24	2.10E-19	8.56E-06	-5.13	3.07E-19	1.04E-05	-5.53	2.65E-19	2.37E-07	2.72
4	Tetrahydrodipicolinate	154.04	11.35	HMDB12289	0.35	P^b >0.05	1.64E-03	0.2	P^b >0.05	1.54E-03	1.16	8.58E-12	7.31E-06	1.13	1.50E-11	2.41E-06	3.91
5	Taurocholic acid 3-sulfate	640.26	10.77	HMDB02581	0.05	2.03E-10	9.85E-04	0	2.30E-10	9.65E-04	0.22	P^b >0.05	4.32E-05	-0.21	3.13E-09	7.17E-05	3.39
6	Sulfolithocholylglycine	512.27	6.08	HMDB02639	3.78	P^b >0.05	3.06E-04	3.55	P^b >0.05	8.84E-04	3.66	P^b >0.05	1.22E-04	5.40	7.65E-03	3.64E-04	13.55
7	Stearoylcarnitine	410.38	11.7	HMDB00848	5.04	2.44E-12	8.79E-06	5.09	1.93E-12	8.77E-06	4.71	1.84E-11	8.53E-06	4.49	P^b >0.05	8.64E-06	3.20
8	Sphinganine	302.31	8.96	HMDB00269	-0.7	2.95E-11	P^c >0.05	-0.74	6.15E-11	P^c >0.05	-0.68	1.02E-08	P^c >0.05	-0.64	1.71E-08	2.25E-02	3.91
9	SM(d18:1/24:1(15Z))	857.67	12.94	HMDB12107	-0.31	3.38E-12	6.03E-03	-0.36	6.24E-12	6.90E-03	-0.42	6.95E-13	3.79E-03	-0.33	1.07E-12	1.67E-02	5.42
10	S-(PGJ2)-glutathione	640.29	12.23	HMDB13063	-0.95	1.89E-13	3.53E-06	-1.01	3.18E-13	3.52E-06	-1.52	3.64E-11	3.84E-06	-1.49	P^b >0.05	3.52E-06	9.62
11	PG(18:3(6Z,9Z,12Z)/18:3(6Z,9Z,12Z))	789.45	4.33	HMDB10666	1.27	9.81E-05	P^c >0.05	1.29	6.67E-05	P^c >0.05	-3.32	2.70E-06	4.19E-08	-1.75	4.96E-05	3.57E-08	2.24
12	PC(18:1(11Z)/20:5(5Z,8Z,11Z,14Z,17Z))	850.56	12.96	HMDB08083	0.7	P^b >0.05	8.90E-06	0.72	P^b >0.05	1.12E-05	-0.45	P^b >0.05	2.51E-05	-2.05	3.61E-03	1.64E-04	5.45
13	Nonate	187.1	4.46	HMDB11717	-1.28	8.20E-15	1.54E-03	-1.31	2.15E-14	1.08E-10	-0.48	3.65E-08	5.95E-05	-0.85	5.43E-11	8.09E-05	3.99
14	N-Acetyl-L-methionine	192.07	6	HMDB11745	-4.92	3.86E-19	P^c >0.05	-4.92	1.09E-18	P^c >0.05	-5.07	1.14E-18	1.03E-07	-5.27	1.05E-18	2.01E-07	2.33
15	N-Acetylgalactosamine	244.08	0.63	HMDB00212	-1.45	1.17E-10	9.99E-07	-1.56	1.97E-10	8.23E-07	-1.46	1.52E-09	1.53E-06	-1.81	2.27E-10	1.31E-06	4.55
16	Myo-inositol hexakisphosphate	682.87	4.75	HMDB03502	-7.55	6.03E-18	3.73E-02	-7.55	1.66E-17	4.58E-02	-3.62	1.05E-16	1.08E-02	-2.97	3.25E-16	4.22E-03	2.24
17	Muricholic acid	453.28	7	HMDB00865	1.66	P^b >0.05	P^c >0.05	1.62	P^b >0.05	P^c >0.05	2.57	3.53E-03	P^c >0.05	2.49	5.34E-03	7.30E-02	7.10
18	Methylglutaric acid	169.04	0.92	HMDB00422	-3.79	9.18E-21	P^c >0.05	-3.82	2.60E-20	P^c >0.05	-3.96	2.88E-20	P^c >0.05	-4.25	2.24E-20	1.73E-02	8.34
19	LysoPC(20:4(5Z,8Z,11Z,14Z))	544.34	10.2	HMDB10395	-0.17	1.70E-03	5.93E-03	-0.15	3.65E-03	4.75E-03	-0.82	1.28E-04	9.13E-05	-1.29	6.45E-06	2.82E-03	16.88
20	LysoPC(18:2(9Z,12Z))	520.34	10.2	HMDB10386	0.17	P^b >0.05	1.39E-04	0.13	8.34E-02	2.74E-04	0.31	P^b >0.05	5.89E-04	-0.178	4.27E-02	1.68E-04	9.48
21	LysoPC(18:0)	524.37	12.22	HMDB10384	-0.54	1.46E-05	3.20E-04	-0.52	3.68E-05	9.11E-04	-0.98	6.75E-06	1.28E-06	-0.50	3.37E-04	4.65E-05	27.20
22	LysoPC(17:0)	508.34	12.23	HMDB12108	-0.79	1.11E-12	6.17E-10	-0.82	2.86E-12	5.10E-09	-1.63	2.47E-14	5.37E-09	-1.66	2.09E-14	4.95E-09	5.77
23	LysoPC(15:0)	482.32	10.07	HMDB10381	0.58	P^b >0.05	1.74E-02	0.56	P^b >0.05	1.73E-02	0.56	5.45E-01	1.97E-02	1.83	1.46E-05	2.02E-02	4.85
24	L-Valine	118.09	0.65	HMDB00883	-0.25	1.24E-02	2.39E-03	-0.41	8.18E-03	1.25E-03	0.88	1.85E-03	2.47E-04	1.31	1.46E-07	4.08E-04	3.05
25	L-Tryptophan	205.1	2.27	HMDB00929	1.21	8.21E-04	3.40E-04	1.16	1.79E-03	3.16E-04	1.75	9.35E-12	5.60E-04	0.96	5.66E-04	3.78E-04	5.21

26	L-Leucine	132.1	1.3	HMDB00687	-0.1	1.04E-03	3.35E-04	-0.22	6.46E-04	2.87E-04	1.09	2.94E-07	6.89E-04	0.93	1.41E-05	4.05E-04	4.62
27	Lithocholyltaurine	528.31	10.2	HMDB00722	-0.59	1.13E-06	P^c >0.05	-0.58	3.10E-06	P^c >0.05	-2.31	9.79E-10	4.03E-02	-2.76	P^b >0.05	9.65E-04	3.26
28	Leukotriene C5	668.29	12.23	HMDB12993	-0.46	6.04E-15	P^c >0.05	-0.51	1.21E-14	P^c >0.05	-0.25	5.53E-09	1.73E-03	-0.56	7.42E-13	1.89E-02	6.19
29	L-Carnitine	162.11	0.63	HMDB00062	-0.98	2.57E-09	1.42E-07	-1.05	4.14E-09	7.22E-07	-0.92	1.31E-07	3.96E-05	-1.16	1.15E-08	3.47E-06	2.99
30	Lactosylceramide (d18:1/12:0)	806.57	12.79	HMDB04866	1.17	P^b >0.05	1.36E-02	1.39	4.03E-02	3.70E-02	-0.35	P^b >0.05	2.34E-02	-1.18	P^b >0.05	1.42E-01	3.87
31	L-Acetylcarnitine	204.12	0.9	HMDB00201	-0.81	2.61E-04	2.96E-02	-0.87	3.66E-04	P^c >0.05	-1	5.27E-04	1.93E-03	-0.99	5.65E-04	1.50E-02	2.64
32	Kinetensin 4-7	616.29	10.2	HMDB12986	-3.42	1.47E-20	3.65E-09	-3.58	3.63E-20	2.58E-08	-3.61	4.50E-20	4.32E-04	-0.89	7.18E-14	8.19E-07	3.12
33	Indoxyl sulfate	212	4.34	HMDB00682	2.24	1.00E-05	8.20E-04	2.14	3.74E-05	5.74E-04	1.02	P^b >0.05	1.86E-02	1.45	5.37E-03	3.13E-04	2.08
34	Hippuric acid	178.05	2.9	HMDB00714	2	5.13E-02	1.29E-03	1.96	5.96E-02	5.36E-03	1.82	4.94E-02	2.63E-04	1.95	2.54E-02	8.09E-04	2.52
35	Glycyl-Phenylalanine	223.11	1.15	HMDB28848	1.7	8.20E-03	P^c >0.05	1.59	1.87E-02	P^c >0.05	2.26	2.56E-07	6.72E-05	2.83	1.64E-11	1.07E-05	2.33
36	Glycocholic acid	464.3	6.76	HMDB00138	1.74	P^b >0.05	P^c >0.05	1.52	P^b >0.05	P^c >0.05	2.18	4.54E-03	P^c >0.05	2.25	1.59E-03	4.72E-02	5.35
37	D-Urobilinogen	1179.6	10.2	HMDB04158	0.33	P^b >0.05	5.15E-07	0.3	P^b >0.05	1.91E-06	-0.31	2.37E-02	9.71E-07	-1.47	2.64E-05	1.05E-06	4.73
38	D-Mannose	225.06	0.63	HMDB00169	0.38	P^b >0.05	1.45E-10	0.38	P^b >0.05	9.04E-10	-0.27	1.64E-04	7.00E-06	-0.33	5.18E-05	8.13E-09	2.97
39	Cholesterol	369.35	11.67	HMDB00067	3.3	2.33E-07	5.84E-09	3.5	2.41E-08	2.27E-08	3.27	2.05E-08	1.26E-06	2.81	9.55E-06	9.58E-07	15.38
40	Adrenochrome o-semiquinone	182.08	1.14	HMDB12883	1.11	8.26E-04	4.94E-07	1.06	1.61E-03	2.30E-06	1.17	9.10E-07	1.20E-06	1.31	2.62E-08	6.54E-06	3.82
41	9,12,13-TriHOME	329.23	6.52	HMDB04708	-2.3	9.61E-15	2.53E-07	-2.33	2.46E-14	4.44E-06	-1.52	3.80E-12	P^c >0.05	-1.77	8.26E-13	1.39E-05	3.69
42	6-Hydroxymelatonin	497.24	3.39	HMDB04081	-0.67	1.29E-08	3.69E-07	-0.68	3.14E-08	3.26E-07	-3.63	7.16E-13	6.44E-07	-4.03	4.88E-13	1.51E-06	2.68
43	3-Oxohexanoic acid	129.05	3.22	HMDB10717	2.17	3.95E-05	2.83E-03	2.06	1.49E-04	3.15E-03	1.39	1.84E-02	2.49E-04	1.61	1.64E-03	2.36E-04	2.06
44	3-Methylhistidine	192.07	2.2	HMDB00479	-4.55	4.03E-18	P^c >0.05	-4.56	1.12E-17	P^c >0.05	-4.99	1.04E-17	3.04E-02	-5.02	1.02E-17	3.44E-04	2.68
45	3-Methylene-indolenine	152.05	12.13	HMDB11664	0.38	P^b >0.05	1.35E-03	0.23	P^b >0.05	1.78E-03	1.17	3.46E-12	7.28E-05	1.16	3.03E-12	5.77E-03	21.89
46	2-Phenylethanol glucuronide	297.1	4.54	HMDB10350	1.59	P^b >0.05	P^c >0.05	1.45	P^b >0.05	P^c >0.05	1.51	2.40E-02	8.77E-04	1.74	3.24E-03	9.28E-04	2.82

[a] Fold change was calculated as a binary logarithm of the average mass response (normalized peak area) ratio between the model group vs the control group, where a positive value means that the average mass response of the metabolite in each group is larger than that in the control group. [b] The P-value was calculated from ANOVA. [c] The P-value was calculated from Student's t-test. [d] VIP was obtained from the PLS-DA model. RT represented Retention Time.

Table S3 Location-based metabolite sets for liver fibrosis from urine and serum Metabolomics Pathway Analysis

NO.	Pathway	Total	Hits	Metabolites	Raw p	-Log(p)	Impact
**urine**							
1	Tryptophan metabolism	79	5	L-Tryptophan; Kynurenic acid; 5-Hydroxyindoleacetylglycine; 3-Methyldioxyindole; 4-(2-Amino-3-hydroxyphenyl)-2,4-dioxobutanoic acid	0.00	7.70	0.11
2	Citrate cycle (TCA cycle)	20	2	cis-Aconitic acid; Isocitric acid	0.01	4.38	0.11
3	Valine, leucine and isoleucine degradation	40	2	L-Leucine; Methylmalonic acid	0.05	3.07	0.02
4	Glyoxylate and dicarboxylate metabolism	50	2	cis-Aconitic acid;Isocitric acid	0.07	2.67	0.03
**serum**							
1	Sphingolipid metabolism	25	3	Sphinganine; Lactosylceramide; Sphingomyelin(SM)	0.00	5.90	0.15
2	Valine, leucine and isoleucine biosynthesis	27	2	L-Valine; L-Leucine	0.04	3.26	0.03
3	Glycerophospholipid metabolism	39	2	LysoPC(17:0); PC(18:1(11Z)/20:5(5Z,8Z,11Z,14Z,17Z	0.07	2.60	0.10
4	Valine, leucine and isoleucine degradation	40	2	L-Leucine; L-Valine	0.08	2.56	0.02
5	Primary bile acid biosynthesis	47	2	Cholesterol; Glycocholic acid	0.10	2.28	0.06
6	Tryptophan metabolism	79	2	L-Tryptophan;6-Hydroxymelatonin	0.23	1.45	0.11
7	Aminoacyl-tRNA biosynthesis	75	3	L-Valine; L-Leucine; L-Tryptophan	0.05	2.91	0.00

Table S4. ROC curves for the diagnosis of liver fibrosis based on the potential biomarkers of the urine and serum samples at week 8.

No.	Metabolites	AUC	Sensitivity	1-Specificity	95%CI
urine					
1	L-Tryptophan	1.00	1.00	0.00	1.00-1.00
2	Kynurenic acid	1.00	1.00	0.00	1.00-1.00
3	5-Hydroxyindoleacetylglycine	1.00	1.00	0.00	1.00-1.00
4	3-Methyldioxyindole	1.00	1.00	0.00	1.00-1.00
5	4-(2-Amino-3-hydroxyphenyl)-2,4-dioxobutanoic acid	0.88	1.00	0.14	0.657-1.105
6	cis-Aconitic acid	1.00	1.00	0.00	1.00-1.00
7	Isocitric acid	1.00	1.00	0.00	1.00-1.00
8	L-Leucine	1.00	1.00	0.00	1.00-1.00
9	Methylmalonic acid	1.00	1.00	0.00	1.00-1.00
serum					
1	Sphinganine	1.00	1.00	0.00	1.00-1.00
2	Lactosylceramide	0.98	1.00	0.14	0.904-1.049
3	L-Valine	1.00	1.00	0.00	1.00-1.00
4	L-Leucine	1.00	1.00	0.00	1.00-1.00
5	L-Tryptophan	1.00	1.00	0.00	1.00-1.00
6	LysoPC(17:0)	1.00	1.00	0.00	1.00-1.00
7	PC(18:1(11Z)/20:5(5Z,8Z,11Z,14Z,17Z))	0.88	1.00	0.14	0.657-1.105
8	Cholesterol	1.00	1.00	0.00	1.00-1.00
9	Glycocholic acid	0.90	0.83	0.14	0.737-1.073
10	Sphingomyelin (d18:1/24:1(15Z))	1.00	1.00	0.00	1.00-1.00

Table S5. ROC curves for the diagnosis of liver fibrosis based on the potential biomarkers of the urine and serum samples at week 2.

No.	Metabolites	AUC	Sensitivity	1-Specificity	95%CI
urine					
1	Kynurenic acid	1.00	1.00	0.00	1.00-1.00
2	5-Hydroxyindoleacetylglycine	1.00	1.00	0.00	1.00-1.00
3	3-Methyldioxyindole	1.00	1.00	0.00	1.00-1.00
4	4-(2-Amino-3-hydroxyphenyl)-2,4-dioxobutanoic acid	0.95	1.00	0.14	0.839-1.066
5	cis-Aconitic acid	0.90	0.83	0.14	0.715-1.094
serum					
1	Sphinganine	1.00	1.00	0.00	1.00-1.00
2	Sphingomyelin	1.00	1.00	0.00	1.00-1.00
3	L-Leucine	0.86	1.00	0.14	0.579-1.116
4	L-Tryptophan	0.97	1.00	0.14	0.886-1.057
5	LysoPC(17:0)	1.00	1.00	0.00	1.00-1.00

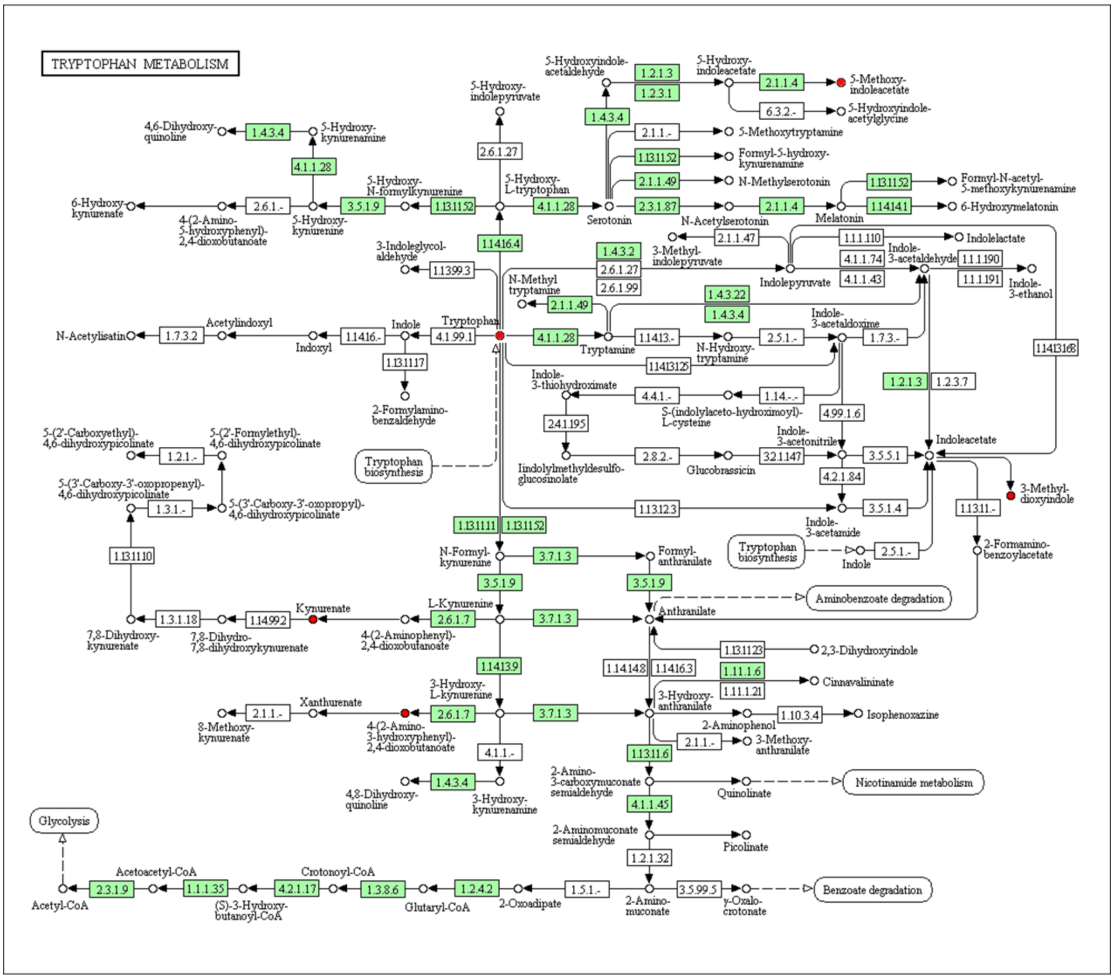

Figure S3. Tryptophan metabolism pathways based on the KEGG database. (Metabolites in red represent the identified potential biomarker in this study)

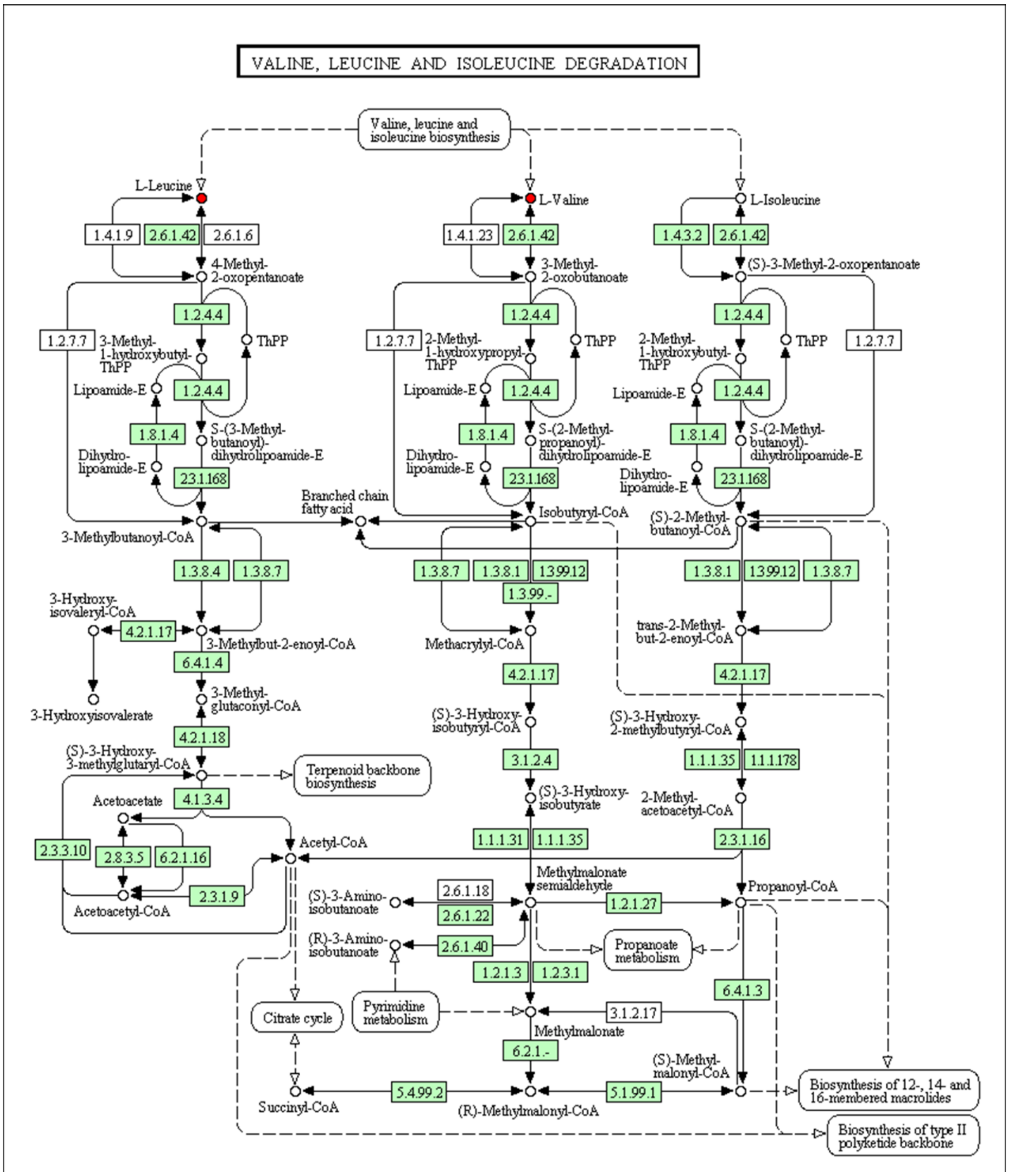

Figure S4. Valine, leucine and isoleucine degradation pathways based on the KEGG database. (Metabolites in red represent the identified potential biomarker in this study)

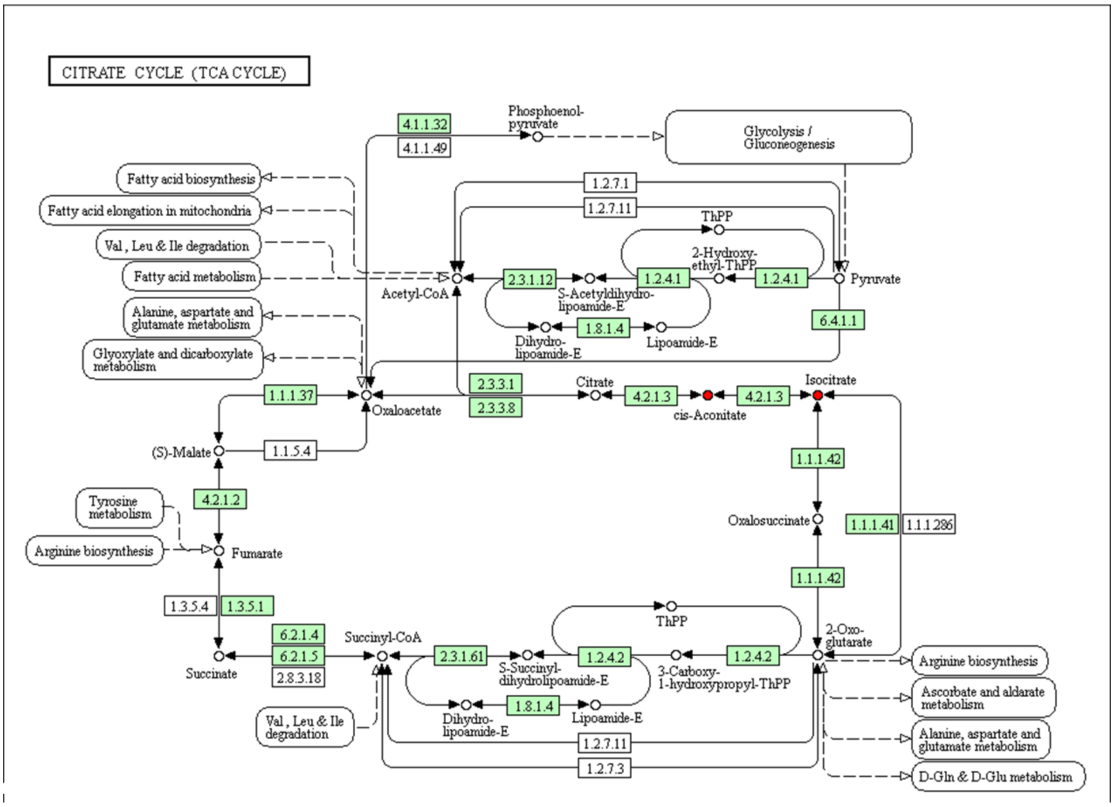

Figure S5. Citrate cycle (TCA cycle) pathways based on the KEGG database. (Metabolites in red represent the identified potential biomarker in this study)

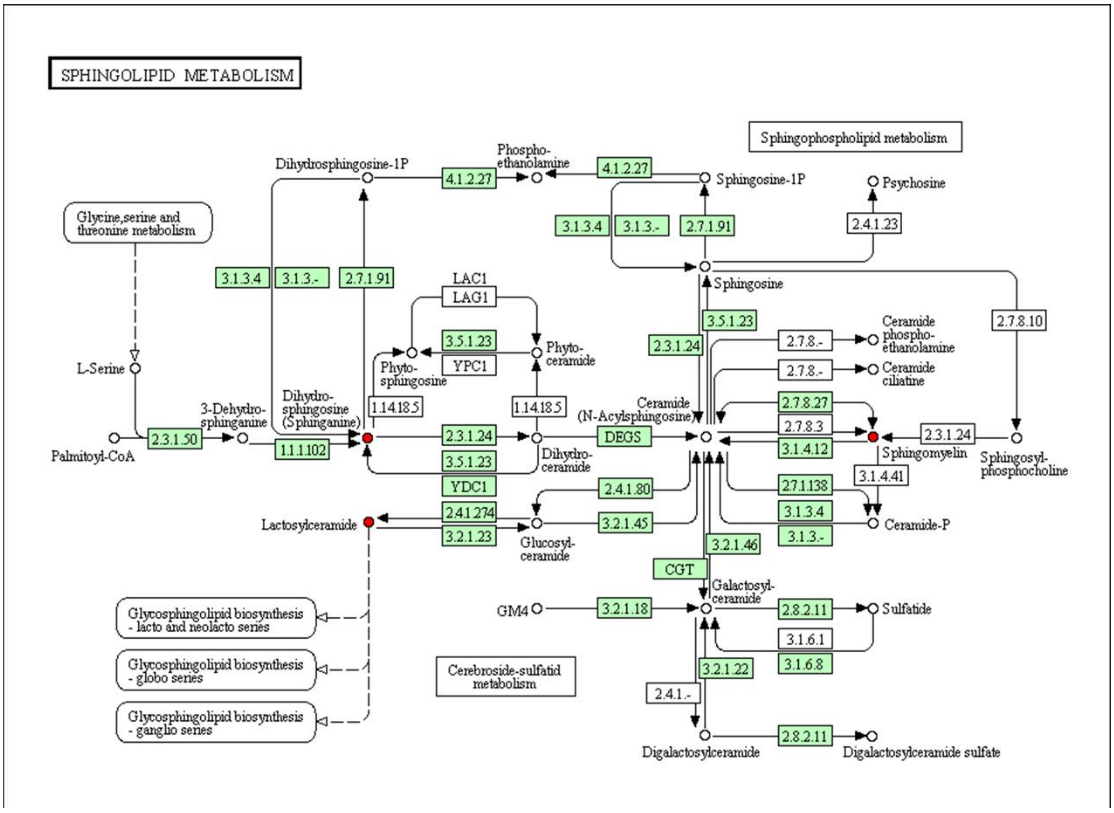

Figure S6. Sphingolipid metabolism pathways based on the KEGG database. (Metabolites in red represent the identified potential biomarker in this study)
